# Rod photoreceptors have a dual dependency on both aerobic glycolysis and OXPHOS and diverge metabolically from other retinal neurons

**DOI:** 10.1126/sciadv.aeg8687

**Published:** 2026-10-07

**Authors:** Gabriele M. Wögenstein, Luca Ravotto, Vyara Todorova, Rachel M. Meister, Marijana Samardzija, L. Felipe Barros, Bruno Weber, Christian Grimm

**Affiliations:** ^1^ Laboratory for Retinal Cell Biology, Department of Ophthalmology, University Hospital Zurich, University of Zurich, Wagistrasse 14, Schlieren, 8952, Zurich, Switzerland.; ^2^ Institute of Pharmacology and Toxicology and Neuroscience Center Zurich, University and ETH Zurich, Winterthurerstr. 190, 8057, Zurich, Switzerland.; ^3^ Centro de Estudios Cientificos-CECs, Valdivia, Chile.; ^4^ Facultad de Medicina, Universidad San Sebastián, Valdivia, Chile.

## Abstract

The retina metabolizes glucose into lactate, a hallmark of aerobic glycolysis known as the Warburg effect. Although evidence points to rod photoreceptors as the primary source of aerobic glycolysis, a comparison of the energy metabolism in different retinal neurons has yet to be performed. We combined two-photon fluorescence lifetime imaging of biosensors with pharmacological protocols to analyze metabolic dynamics in healthy and diseased rod photoreceptors and RBPMS-positive ganglion and calretinin-positive amacrine cells. Our data reveal distinct metabolic profiles among retinal neurons, identify rods as the drivers of aerobic glycolysis, demonstrate that inner retinal neurons rely on oxidative phosphorylation, show that rods need both glycolysis and oxidative phosphorylation to maintain ATP levels, and suggest that rods can metabolize lactate. A mutation causing retinitis pigmentosa increases lactate production in rods but changes the energy metabolism only subtly otherwise. Our results improve the understanding of retinal physiology and are relevant for pathologies involving imbalanced energy metabolism.

## INTRODUCTION

Retinal cells convert light into electrochemical signals that are relayed to the brain via the optic nerve. As these processes are energetically costly, the retina has a very high metabolic demand (*1*, *2*). More than a century ago, Otto Warburg observed that the retina, similar to tumor slices, metabolize glucose to lactate even in the presence of oxygen—a phenomenon known as aerobic glycolysis, which was later named after its discoverer (*3*, *4*).

The integration of experimental data with theoretical models has led to the concept of a metabolic ecosystem in the outer retina (*5*). According to this model, glucose is transported from the choroid across the retinal pigment epithelium (RPE) to photoreceptors (*6*). In photoreceptors, glucose supports the generation of biosynthetic intermediates required for photoreceptor outer segment renewal and serves as substrate for the production of ATP and lactate through aerobic glycolysis (*4*, *7*, *8*). The produced lactate is subsequently exported and taken up by the RPE, where it fuels oxidative phosphorylation (OXPHOS) (*5*, *9*). The concept of a metabolic ecosystem is supported by the expression of key glycolytic enzyme isoforms in rods and further reinforced by studies showing that reduced expression of enzymes such as hexokinase 2 (HK2), lactate dehydrogenase (LDH) A, and pyruvate kinase M2 (PKM2) compromises photoreceptor structure and function (*10*–*15*). In contrast to photoreceptors, neurons of the inner retina, including bipolar, horizontal, amacrine, and ganglion cells, are thought to rely predominantly on OXPHOS for their function, reflecting their distinct metabolic needs (*16*–*19*). These differences in metabolic requirements across retinal cell classes likely arise from their specialized functions, position in retinal circuitry, and differential access to blood supply. Inner retinal cells are supplied by three vascular plexi that provide a relatively stable source of oxygen and nutrients. Photoreceptors depend on the diffusion or transport of molecules from the choroidal vasculature (*20*) that can be affected by disease or age. Therefore, retinal metabolism is thought to be specialized and heterogeneous across cell classes.

Most conclusions regarding retinal energy metabolism are based on genetic models, on protein and gene expression data, or on ^13^C tracing experiments using whole retinas (*9*–*15*, *19*, *21*–*25*). Only a few studies measured metabolites in specific retinal cell classes directly (*26*–*29*). Therefore, assessments of retinal metabolism often lack cellular resolution, offering only limited data for a direct comparison between inner and outer retinal neurons. To overcome this limitation, we used two-photon fluorescence lifetime imaging microscopy (2P-FLIM) in combination with the cell-type-specific expression of genetically encoded biosensors to study energy metabolism on a cell-type-specific level. Because the fluorescence lifetime is an intrinsic property of a given fluorophore, 2P-FLIM permits the evaluation and comparison of metabolite concentrations in different cell classes (*30*). When combined with pharmacological protocols, 2P-FLIM thus becomes a powerful tool to investigate cellular energy metabolism. Using this approach, we revealed the differential dynamics in the ATP and lactate metabolism in rods in the outer retina and in RNA binding protein with multiple splicing (RBPMS) positive ganglion and calretinin-positive amacrine cells in the inner retina, in healthy mice and in a model of retinal degeneration.

## RESULTS

### Two-photon laser scanning (2P) microscopy triggers Ca^2+^ signaling in rod photoreceptors in acute eye sections

The retina contains five classes of neurons that process visual information, plus three classes of glia cells that support and complement the neurons. Ganglion cells, amacrine cells, bipolar cells, and horizontal cells are located in the inner retina, as are astrocytes, microglia and Müller glia cells. Photoreceptors are found in the outer retina (*31*). The metabolic ecosystem in the outer retina relies on the exchange of metabolites between photoreceptors and the adjacent RPE cells, suggesting that the two cell classes form a functional unit. To visualize the integrity of this RPE/photoreceptor unit in our acute eye sections and to assess photoreceptor function, we subretinally injected a mixture of AAVs into wild type (BALB/c) mice. These AAVs encoded mCherry under the vitelliform macular dystrophy 2 (VMD2) promoter for RPE-specific expression (*32*) and the calcium sensor GCaMP6s under the control of the mouse opsin promoter (mOP) for rod-specific expression (Fig. 1A) (*26*, *33*). The albinotic mice were chosen for this experiment because they lack pigmentation, which allowed the visualization of mCherry expressing RPE cells using 2P-microscopy. Imaging of acute eye sections four weeks after the injection confirmed expression of mCherry in RPE cells and GCaMP6s in adjacent rods, revealing the integrity of the RPE/photoreceptor unit (Fig. 1B). Next, we evaluated the functionality of the rods by measuring intracellular Ca^2+^ dynamics in response to light pulses using 2P microscopy of GCaMP6s (*34*). Activation of the phototransduction cascade by light results in the closure of the cGMP-gated ion channels and a subsequent decrease in cytosolic Ca^2+^ levels (*35*). This is reflected by a reduction in the fluorescence signal intensity of the sensor (Fig. 1C). Since the discrimination between outer and inner segments was not possible, we analyzed the changes in the GCaMP6s fluorescence signal in the entire photoreceptor segments. The reduction of the signal intensity during the 10-second recording interval (Fig. 1, D and E) revealed a repetitive decrease in intracellular Ca^2+^ levels and thus the presence of functional, light-responsive rods in our sections. This response was likely triggered by the light emitted from activated sensors, which falls within the range of the absorption maximum of rhodopsin (*36*–*39*), or by direct activation through the short infrared light pulses as previously described (*40*). The light-induced drop in calcium was reversed during a 30-second interval in darkness even across multiple imaging and recovery cycles (Fig. 1E), indicating that the system was stable and functional. This was further supported by the slow increase of Ca^2+^ levels in the presence of the non-cleavable cGMP analog 8-pCPT-cGMP to reopen the channels during imaging (Fig. 1F) (*41*). The delay in the response of approximately 100 seconds corresponded to the time required for 8-pCPT-cGMP to reach the imaging chamber (fig. S1A). Together, these data verified that rods were fully functional in the acute eye sections and responded to 2P by activating phototransduction (*26*, *38*). Therefore, all subsequent data generated by 2P-FLIM represent conditions in light-exposed rods.

**Fig. 1.**
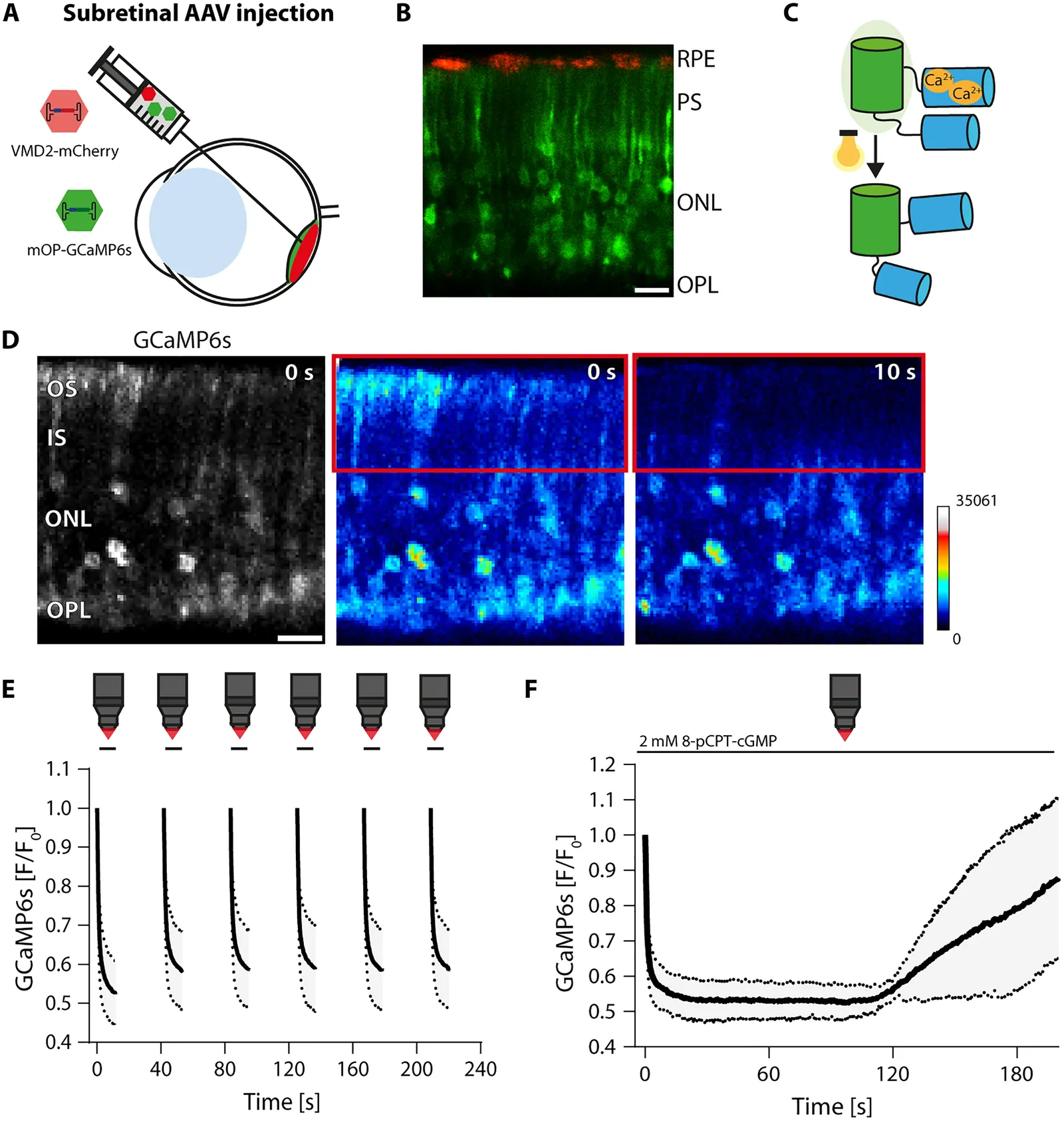
Light-induced calcium response in rods of wildtype mice (BALB/c) during 2P. (**A**) Schematic of subretinal injection delivering a mixture of two AAV vectors: one encoding mCherry under the VMD2 promoter and another encoding the calcium sensor GCaMP6s under the mOP promoter. (**B**) Representative 2P image of an acute eye section from a mouse injected with the AAV mix shown in (A). RPE cells express mCherry (red) and rod photoreceptors GCaMP6s (green). Scale bar: 10 μm. (**C**) Schematic of GCaMP6s (*102*). Fluorescence intensity of circularly permuted green fluorescent protein (cpGFP) increases when Ca^2+^ is bound. Light-driven reduction in Ca^2+^ in rods, results in the decrease of fluorescence intensity. (**D**) Representative 2P images of rod photoreceptors expressing GCaMP6s. Maximum intensity image at 0 s (left) and intensity weighted images at 0 s (middle) and 10 s (right) of imaging. Red boxes mark the analyzed area (photoreceptor segments). Scale bar: 10 μm. (**E**) GCaMP6s traces (mean ± SD) repetitively imaged in photoreceptor segments of wildtype mice (*N* = 5, *n* = 3) during 10 s of 2P imaging followed by 30 s recovery. (**F**) GCaMP6s traces (mean ± SD) imaged in the photoreceptor segments of wildtype mice (*N* = 3, *n* = 3) in the presence of the cGMP analog (2 mM 8-pCPT-cGMP). RPE, retinal pigment epithelium; OS, outer segments; IS, inner segments; PS, photoreceptor segments; ONL, outer nuclear layer; OPL, outer plexiform layer.

### Rods rely on glucose whereas inner retinal neurons can use lactate in the absence of glucose to fully maintain ATP levels

To determine the energy source that fuels ATP production in the different neuronal classes, we delivered the ATP sensor ATeam1.03 (*42*) (Fig. 2A) to rods in the outer retina and to RBPMS-positive ganglion and calretinin-positive amacrine cells, further referred to as “inner retinal neurons,” via subretinal or intravitreal AAV injections (fig. S1, B and C). The fluorescence lifetime (τ_MC_) of the sensor expressed in rods was stable for at least 25 min during the “control” protocol and increased (indicating a reduction in ATP levels) only after removal of glucose and application of the reversible OXPHOS blocker NaN_3_ (sodium azide) (fig. S2A) (*43*). The recovery of the ATP signal upon NaN_3_ washout confirmed the viability of the cells during the duration of the protocol (fig. S2B) and further validated our setup.

**Fig. 2.**
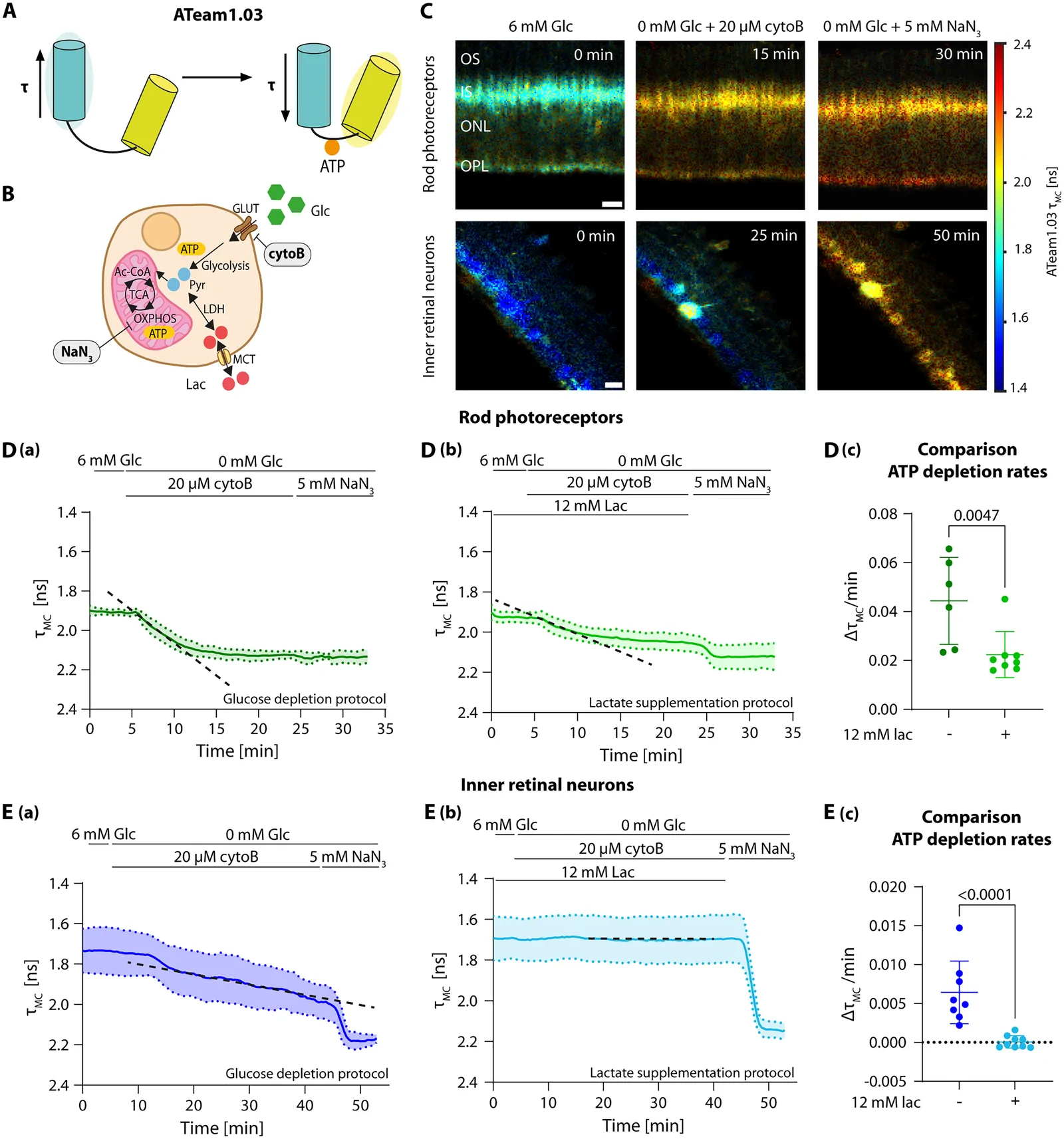
ATP dynamics in rods and inner retinal neurons of wildtype mice (129S6). (**A**) Schematic representation of the ATP sensor ATeam1.03 (*42*). Binding of ATP decreases the fluorescence lifetime (τ). (**B**) Schematic representation of cellular metabolism and the drugs used during the glucose depletion and lactate supplementation: GLUTs were blocked with cytoB and OXPHOS with NaN_3_ (Table 1) during aglycemia. (**C**) 2P-FLIM images (τ_MC_) of ATeam1.03 in rods (upper panel) and inner retinal neurons (lower panel) at different time points during glucose depletion. Scale bars: 10 μm. [**D**(**a**) and **E**(**a**)] Lifetime traces of ATeam1.03 in rods [D(a); *N* = 6, *n* = 6] and inner retinal neurons [E(a); *N* = 8, *n* = 6] during glucose depletion (mean ± SD). Black dashed lines: slopes used to calculate ATP depletion rates. [**D**(**b**) and **E**(**b**)] Lifetime traces of ATeam1.03 in rods [D(b); *N* = 8, *n* = 5] and inner retinal neurons [E(b); *N* = 9, *n* = 5] during lactate supplementation (mean ± SD). Black dashed lines: slopes used to calculate ATP depletion rates. [**D**(**c**) and **E**(**c**)] Comparison of the ATP depletion rates (∆τ_MC_/min) during the glucose depletion (dark green, dark blue) and lactate supplementation (light green, light blue) in rods [D(c)] and inner retinal neurons [E(c)]. Shown are individual values and means ± SD. Mann-Whitney nonparametric test. OS, outer segments; IS, inner segments; ONL, outer nuclear layer; OPL, outer plexiform layer.

We used “glucose depletion” (Table 1) to investigate changes of intracellular ATP levels in glucose-free conditions. Apart from glucose removal from the medium, this protocol included inhibition of glucose transporters (GLUTs) 1, 3 and 4 with cytochalasin B (cytoB) (*44*–*46*) and a subsequent OXPHOS block with NaN_3_ (Fig. 2B). In rods, glucose-free conditions induced a rapid drop of ATP to low steady-state levels, which were not further reduced by blocking OXPHOS with NaN_3_ [Fig. 2, C and D(a)]. Low ATP levels after glucose withdrawal rapidly recovered to basal levels after re-addition of glucose, indicating that the energy production capacity was not impaired by the transient absence of the substrate (fig. S2C).

**Table 1.** Pharmacological protocols for 2P/2P-FLIM.

Protocol name	Protocol	Sensor
Control	23.5 min ACSF / 6 mM D-glucose ➔ 10 min 0 mM D-glucose + 6 mM sucrose + 5 mM NaN_3_	ATeam1.03
NaN_3_ washout	3.5 min ACSF / 6 mM D-glucose ➔ 10 min 6 mM D- glucose + 5 mM NaN_3_ ➔ 16.5 min 6 mM D-glucose	ATeam1.03
Glucose depletion	3.5 min ACSF / 6 mM D-glucose ➔ 20 (or 40) min 0 mM D-glucose + 6 mM sucrose + 20 μM cytoB ➔ 10 min 0 mM D-glucose + 6 mM sucrose + 5 mM NaN_3_	ATeam1.03
Glucose recovery	3.5 min ACSF / 6 mM D-glucose ➔ 18 min 0 mM D-glucose + 6 mM sucrose ➔ 12 min 6 mM D-glucose	ATeam1.03
Lactate supplementation	3.5 min ACSF / 6 mM D-glucose + 12 mM sodium L-lactate ➔ 20 (or 40) min 0 mM D-glucose + 6 mM sucrose + 12 mM sodium L-lactate + 20 μM cytoB ➔ 10 min 0 mM D-glucose + 6 mM sucrose + 5 mM NaN_3_	ATeam1.03
Glucose-lactate-glucose exchange	3.5 min ACSF / 6 mM D-glucose ➔ 11.5 min 0 mM D-glucose + 6 mM sucrose + 12 mM sodium L-lactate ➔ 25 min 6 mM D-glucose	GCaMP6s
Glutamine supplementation	3.5 min ACSF / 6 mM D-glucose + 6 mM L-glutamine ➔ 20 min 0 mM D-glucose + 6 mM sucrose + 6 mM L-glutamine + 20 μM cytoB ➔ 10 min 0 mM D-glucose + 6 mM sucrose + 5 mM NaN_3_	ATeam1.03
Lactate transport block	3.5 min ACSF / 6 mM D-glucose ➔ 40 min 0 mM D-glucose + 6 mM sucrose + 20 μM cytoB + 5 μM AR-C155858 ➔ 10 min 0 mM D-glucose + 6 mM sucrose + 5 mM NaN_3_	ATeam1.03
Glycogen phosphorylase block	Prior to imaging the sections were pre-incubated for 1 hour with ACSF containing 300 μM DAB / 6 mM D-glucose	ATeam1.03
	3.5 min ACSF / 6 mM D-glucose + 300 μM DAB ➔ 40 min 0 mM D-glucose + 6 mM sucrose + 20 μM cytoB + 300 μM DAB ➔ 10 min 0 mM D-glucose + 6 mM sucrose + 5 mM NaN_3_	
OXPHOS block followed by aglycemia	3.5 min ACSF / 6 mM D-glucose ➔ 10 min 6 mM D-glucose + 5 mM NaN_3_ ➔ 16.5 min 0 mM D-glucose + 6 mM sucrose + 5 mM NaN_3_	ATeam1.03
Lactate production 1	3.5 min ACSF / 6 mM D-glucose ➔ 15 min 6 mM D-glucose + 5 μM AR-C155858 ➔ 15 min 0 mM D-glucose + 6 mM sucrose + 5 μM AR-C155858	LiLac
Lactate production 2	3.5 min ACSF / 6 mM D-glucose ➔ 15 min 6 mM D-glucose + 5 μM AR-C155858 + 4 μM syrosingopine ➔ 15 min 0 mM D-glucose + 6 mM sucrose + 5 μM AR-C155858 + 4 μM syrosingopine	LiLac
Syrosingopine test	3.5 min ACSF / 6 mM D-glucose ➔ 15 min 6 mM D-glucose + 4 μM syrosingopine ➔ 15 min 0 mM D-glucose + 6 mM sucrose + 4 μM syrosingopine	LiLac

In contrast to rods, inner retinal neurons maintained their baseline ATP levels for almost 10 min before ATP levels slowly declined in the absence of glucose [Fig. 2, C and E(a)]. Of note, even after 40 min without glucose, inner retinal neurons were not fully depleted of ATP, since blocking OXPHOS further decreased ATP levels [Fig. 2E(a)]. This suggests that inner retinal neurons can partially sustain ATP production via OXPHOS in the absence of glucose, potentially by using alternative substrates supplied by neighboring cells. In contrast, rods are glucose-dependent and deplete ATP rapidly under glucose-free conditions.

We used “lactate supplementation” (Table 1) to investigate if lactate could be an alternative source to glucose, especially for inner retinal neurons. In rods, ATP levels dropped after withdrawal of glucose also in the presence of lactate [Fig. 2D(b)], albeit at a slower rate [∆τ_MC_/min, Fig. 2D(c)] and only to intermediate levels. ATP dropped completely only after blocking OXPHOS. Interestingly, this drop of ATP to intermediate levels was accompanied by drastically reduced photoreceptor function, indicated by the strongly reduced decline of intracellular Ca^2+^ levels in response to the 2P-excitation light. Although the function slowly recovered after replacing lactate with glucose, the response did not reach basal levels during the experimental time frame, presumably because the cells still suffered from the stress induced by the transiently reduced ATP levels (fig. S3).

In sharp contrast, lactate supplementation fully maintained baseline ATP levels in inner retinal neurons for at least 40 min and dropped only after blocking OXPHOS [Fig. 2E(b and c)]. This data indicates that inner retinal neurons efficiently produced ATP through OXPHOS when lactate substituted glucose in the medium. In contrast, rods could only partially maintain ATP levels in the presence of lactate alone. Alternative to the direct consumption of lactate by rods, neighboring cells may have potentially converted lactate from the medium to glutamine that was then supplied to rods as fuel (fig. S4A). Indeed, providing glutamine in the medium instead of glucose (“glutamine supplementation,” Table 1) enabled rods to maintain intermediate steady-state ATP levels comparable to lactate supplementation, which dropped further only after blocking OXPHOS (fig. S4, B to D). This shows that rods can use glutamine for ATP production through OXPHOS, as suggested before (*47*–*49*), but cannot maintain baseline ATP levels on glutamine alone.

The rather slow decline of ATP levels in the absence of glucose in inner retinal neurons [Fig. 2E(a)] might indicate the presence of alternative fuels that can be metabolized through OXPHOS in these cells. To test if adjacent cells, such as Müller glia, might release lactate to support glucose-deprived inner retinal neurons, we inhibited monocarboxylate transporter (MCT) 1 and 2 with AR-C155858 (*50*) using the “lactate transport block” protocol (fig. S5A; Table 1). This did not result in a significantly faster ATP decline (fig. S5, B to D) suggesting that monocarboxylates provided by neighboring cells did not significantly impact ATP production in inner retinal neurons. Thus, we tested a potential contribution of glycogen for ATP production in inner retinal neurons in the absence of glucose. Blocking glycogenolysis by inhibiting the glycogen phosphorylase with 1,4-Dideoxy-1,4-imino-D-arabinitol hydrochloride (DAB, fig. S5E) (*51*) resulted in a significantly faster decline of ATP levels (fig. S5, F to H), suggesting that glycogen can serve as an alternative source for ATP in the inner retina.

### Rods are more glycolytic and inner retinal neurons more oxidative

To directly test if rod photoreceptors rely on both OXPHOS and aerobic glycolysis and inner retinal neurons mainly on OXPHOS, we blocked OXPHOS with NaN_3_ in the presence and absence of glucose (“OXPHOS block followed by aglycemia”; Table 1). Blocking OXPHOS in the presence of glucose reduced ATP levels in rods to intermediate levels (Fig. 3, A and B). Only after additional removal of glucose (aglycemia) did ATP levels drop completely showing that rods produced ATP via glycolysis but indeed needed OXPHOS to maintain full ATP levels. By contrast, ATP levels in the inner retinal neurons declined to very low levels after OXPHOS was blocked. The additional removal of glucose had only a marginal additional effect (Fig. 3, A and C) suggesting that aerobic glycolysis contributed minimally, if at all, to ATP production. The direct comparison of slope 1 (Fig. 3D) and slope 2 (Fig. 3E) between rods and inner retinal neurons demonstrated significant differences in ATP production: while rods produced ATP via both aerobic glycolysis and OXPHOS, inner retinal neurons relied almost exclusively on OXPHOS.

**Fig. 3.**
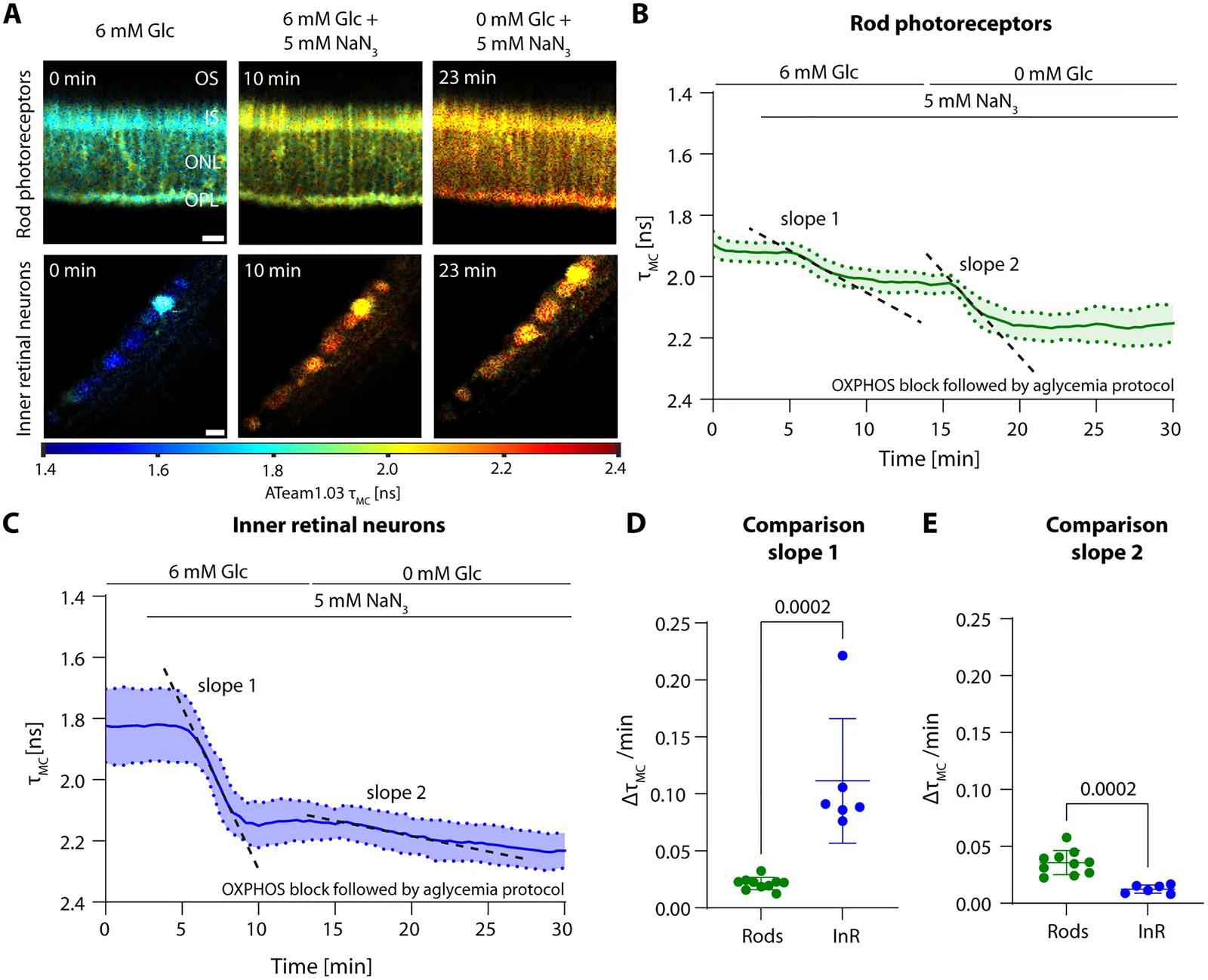
ATP production via aerobic glycolysis versus OXPHOS in rods and inner retinal neurons of wildtype mice (129S6). (**A**) 2P-FLIM images (τ_MC_) of ATeam1.03 in rods (upper panel) and inner retinal neurons (lower panel) at different time points during the OXPHOS block followed by aglycemia (Table 1). Scale bars: 10 μm. (**B** and **C**) Lifetime traces of ATeam1.03 in rods [(B); *N* = 11, *n* = 8] and inner retinal neurons [(C); *N* = 6, *n* = 5] during the OXPHOS block with NaN_3_ followed by aglycemia (0 mM Glc; mean ± SD). Black dashed lines: slopes used to calculate ATP depletion rates. (**D** and **E**) Comparison of ATP depletion rates (∆τ_MC_/min) in rods (green) and inner retinal neurons (blue) after OXPHOS block with NaN_3_ [slope 1, data from (B) and (C)] and subsequent glucose removal [slope 2, data from (B) and (C)]. Shown are individual values and means ± SD. Mann-Whitney nonparametric test. OS, outer segments; IS, inner segments; ONL, outer nuclear layer; OPL, outer plexiform layer.

To further validate that inner retinal neurons are more oxidative than rods, we expressed Peredox, a fluorescent biosensor that reports the cytosolic NADH/NAD^+^ ratio and thus the redox state of the cell (fig. S6A) (*52*, *53*). Cytosolic NADH is primarily generated by glyceraldehyde-3-phosphate dehydrogenase (GAPDH) during glycolysis and must be reoxidized to NAD^+^ for glycolysis to continue, e.g., via mitochondrial shuttles. Since these shuttles are saturated in highly glycolytic cells, NAD^+^ regeneration occurs mainly via LDH activity, leading to a higher NADH/NAD^+^ ratio compared to oxidative cells (fig. S6B) (*54*). The higher fluorescence lifetime of Peredox (high NADH/NAD^+^ ratio) in rods at baseline (fig. S6, C and D) supports our conclusion from the ATP sensor that rod photoreceptors favor aerobic glycolysis, whereas inner retinal neurons rely more on mitochondrial respiration for energy production. Taken together, these data demonstrate that rods use aerobic glycolysis to produce ATP but need OXPHOS in addition to fully maintain their ATP levels. In contrast, inner retinal neurons depend mainly on OXPHOS.

### Rods produce more lactate than inner retinal neurons

Our data indicated that rods are the main cells performing aerobic glycolysis. Therefore, we sought to determine if the retinal neurons differ in their production of lactate, the final product of aerobic glycolysis. To this end, we used LiLac, a single fluorophore biosensor that decreases its fluorescence lifetime upon binding lactate (Fig. 4A) (*55*). Cytosolic lactate levels are influenced by two processes: interconversion with pyruvate via LDH, and exchange with the extracellular space via MCTs (*56*). To clarify the relative contribution of these two pathways, we affected lactate transport by blocking MCT1 and MCT2, the main lactate transporters in photoreceptors and inner retinal neurons (*57*) (Fig. 4B; Table 1 “lactate production 1”). This resulted in higher (Fig. 4, C and D) and faster (Fig. 4, D and E; slope 1) intracellular lactate accumulation in rods than in inner retinal neurons, suggesting stronger lactate production from pyruvate in rods, further supporting the high capacity of rods to perform aerobic glycolysis. After removal of glucose, lactate levels rapidly declined (Fig. 4, D and F; slope 2) in both rod photoreceptors and inner retinal neurons. This drop in the presence of the MCT1/2 inhibitor could be caused by the conversion of lactate to pyruvate by LDH, by loss of lactate through MCT4, which has been reported in rods (*57*), or by an additional lactate permeability. To test a potential contribution of MCT4, we added the MCT1/4 blocker syrosingopine (*27*, *58*) together with the MCT1/2 blocker (fig. S7A; Table 1 “lactate production 2”). This did not alter lactate dynamics compared to inhibiting MCT1/2 alone (fig. S7, B to E), indicating a minor role for MCT4 in the lactate exchange between rods and the extracellular environment. This was further evidenced by the much slower and delayed increase of intracellular lactate when syrosingopine, which has a 60-fold higher potency for blocking MCT4 than for MCT1 (*58*), was used alone (fig. S7, F and G; “syrosingopine test”; Table 1). This showed that the MCT1/4 blocker was functional and supported the conclusion that MCT4 is not a major transporter for lactate in rods. When glucose was withdrawn in the presence of syrosingopine (fig. S7, F and H), however, lactate declined at a rate similar to that observed during the MCT1/2 block again suggesting the conversion of lactate to pyruvate in rods. However, we cannot rule out, that under these conditions, increased membrane permeability led to some lactate leaking out of the cell.

**Fig. 4.**
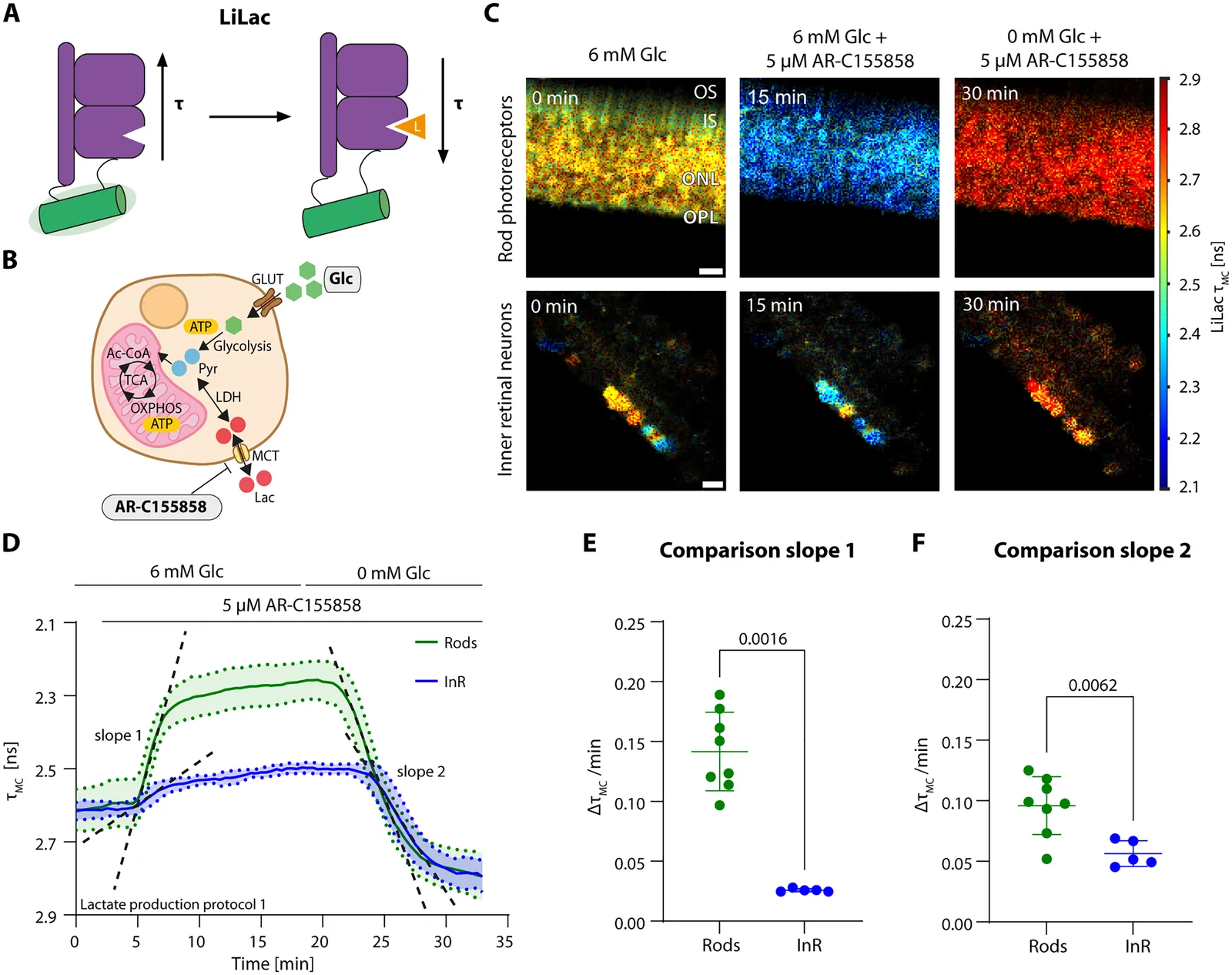
Lactate production in rods and inner retinal neurons of wildtype mice (129S6). (**A**) Schematic representation of the lactate sensor LiLac (*55*). The lifetime (τ) of LiLac decreases when bound to lactate (L). (**B**) Schematic representation of cellular metabolism, illustrating lactate transport via MCTs and their blocking with AR-C155858. (**C**) 2P-FLIM images (τ_MC_) of LiLac in rods (upper panel) and inner retinal neurons (lower panel) at different time points during lactate production 1 (Table 1). Scale bars: 10 μm. (**D**) Lifetime traces (means ± SD) of LiLac in rods (green; *N* = 8, *n* = 3) and inner retinal neurons (blue; *N* = 5, *n* = 3) during lactate production 1. Black dashed lines: slopes used to calculate the changes in the lifetime in LiLac shown in (E) and (F). (**E** and **F**) Lifetime changes (∆τ_MC_/min) in LiLac during the MCT block in the presence of glucose [(E); slope 1; lactate production] and in the absence of glucose [(F); slope 2] in rods (green) and inner retinal neurons (blue). Shown are individual values and means ± SD. Mann-Whitney nonparametric test. OS, outer segments; IS, inner segments; ONL, outer nuclear layer; OPL, outer plexiform layer.

### Energy metabolism in degenerating rods of *Rho*^*P23H/+*^ mice

To assess potential metabolic changes during inherited retinal degeneration, we repeated these experiments in rods of *Rho^P23H/+^* mice, an established model for retinitis pigmentosa (RP) (*59*). The *Rho^P23H/+^* mice were analyzed at 8 weeks of age when the thickness of the outer nuclear layer was already reduced by approximately 50% and photoreceptor segments were disorganized and shortened (fig. S8A) (*59*). To confidently compare the *Rho^P23H/+^* data to wildtype rods, we first evaluated the vital state of the degenerating rods by measuring Ca^2+^ in response to imaging using GCaMP6s (fig. S8B). As observed in wildtype rods (Fig. 1), the Ca^2+^ levels dropped in photoreceptor segments of the *Rho^P23H/+^* mouse during imaging and recovered within 30 s in darkness (fig. S8, C and D). During continuous imaging, a sustained increase in Ca^2+^ was observed following the inflow of 8-pCPT-cGMP (fig. S8E), indicating that the *Rho^P23H/+^* rods were still functional and in a light-activated state during imaging. This implies that a direct comparison of the metabolic dynamics between healthy and diseased rod photoreceptors was possible.

To determine whether degenerating processes changed the ATP dynamics in rods, we applied the glucose depletion and lactate supplementation protocols (Table 1) and compared the data to those from wildtype 129S6 mice generated in Figs. 2 to 4. Similarly to wildtype, the *Rho^P23H/+^* rods showed rapidly reduced ATP levels in the absence of glucose (Fig. 5, A to C) and only partially maintained ATP levels when lactate substituted glucose (Fig. 5D). The decline in ATP levels in the absence of glucose was slowed when lactate was present (Fig. 5E) but was faster than in wildtype rods (Fig. 5F), suggesting that ATP production from lactate via OXPHOS was less efficient in the degenerating rods.

**Fig. 5.**
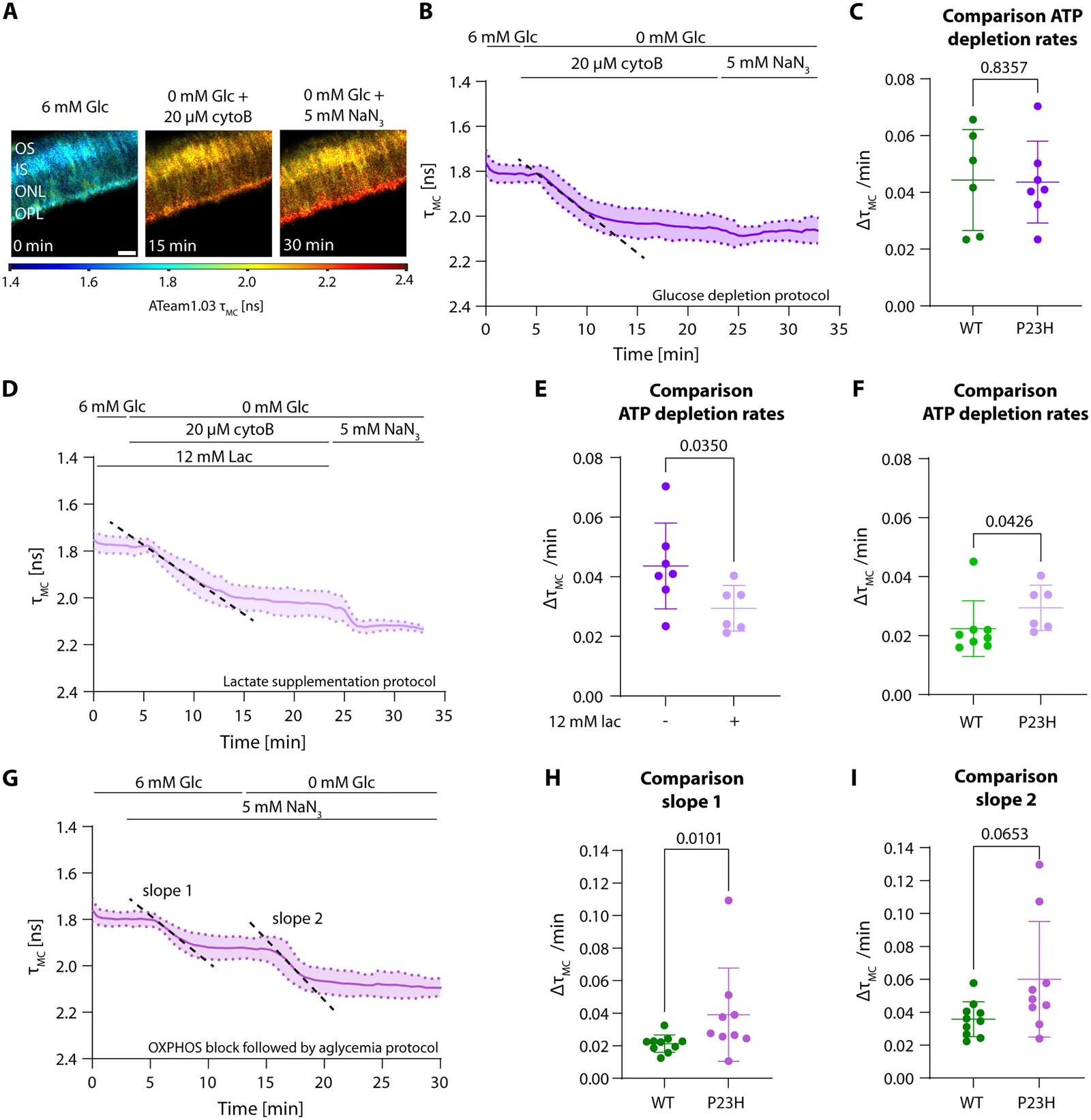
ATP dynamics in degenerating rods of *Rho^P23H/+^* mice. (**A**) 2P-FLIM images (τ_MC_) of ATeam1.03 in rods of *Rho^P23H/+^* mice at different time points during glucose depletion (Table 1) and (**B**) corresponding lifetime trace (mean ± SD; *N* = 7, *n* = 5). Scale bar: 10 μm. Black dashed line: slope used to calculate ATP depletion rates. (**C**) Comparison of ATP depletion rates (∆τ_MC_/min) during glucose depletion in rods of wt [green; data from Fig. 2D(c)] and *Rho^P23H/+^* mice (violet). Shown are individual values and means ± SD. Mann-Whitney nonparametric test. (**D**) Lifetime trace of ATeam1.03 in rods of *Rho^P23H/+^* mice during lactate supplementation 1 (Table 1; mean ± SD; *N* = 6, *n* = 5). Black dashed line: slope used to calculate the ATP depletion rate. (**E**) Comparison of ATP depletion rates (∆τ_MC_/min) during glucose depletion (dark violet) and lactate supplementation 1 (light violet). Shown are individual values and means ± SD. Mann-Whitney nonparametric test. (**F**) Comparison of ATP depletion rates (∆τ_MC_/min) in wt [green; data from Fig. 2D(c)] and *Rho^P23H/+^* mice (light violet) during lactate supplementation 1. Shown are individual values and means ± SD. Mann-Whitney nonparametric test. (**G**) Lifetime trace of ATeam1.03 in rods of *Rho^P23H/+^* mice during OXPHOS block followed by aglycemia (Table 1; mean ± SD; *N* = 9, *n* = 5). Black dashed lines: slopes used to calculate ATP depletion rates. (**H** and **I**) Comparison of ATP depletion rates (∆τ_MC_/min) in wt (green; data from Fig. 3, D and E) and *Rho^P23H/+^* mice (violet) during the OXPHOS block [(H); slope 1] followed by aglycemia [(I); slope 2]. Shown are individual values and means ± SD. Mann-Whitney nonparametric test. OS, outer segments; IS, inner segments; ONL, outer nuclear layer; OPL, outer plexiform layer.

*Rho^P23H/+^* rods also performed both aerobic glycolysis and OXPHOS as indicated by the two-step reduction in ATP levels after OXPHOS block and additional glucose withdrawal (Fig. 5G). However, the initial reduction (slope 1; Fig. 5, G and H) was steeper in degenerating rods, which suggests that blocking OXPHOS had a greater impact on ATP levels than in healthy rods. However, the contribution of aerobic glycolysis to the ATP levels was similar in wildtype and degenerating rods (slope 2; Fig. 5I).

Our results indicated subtle metabolic changes in the use of lactate for ATP production in *Rho^P23H/+^* rods. To directly test lactate production and potential consumption in this model, we expressed LiLac and blocked MCT1/2 in the presence of glucose. Degenerating rods accumulated more lactate under these conditions and did so at a faster rate than wildtype rods (Fig. 6, A to C; slope 1), indicating that ATP production in *Rho^P23H/+^* rods may have shifted slightly towards aerobic glycolysis. The rate of lactate reduction after glucose withdrawal (Fig. 6, B and D; slope 2) and the dependency on glucose as energy source (Fig. 5), however, were similar in normal and degenerating rods.

**Fig. 6.**
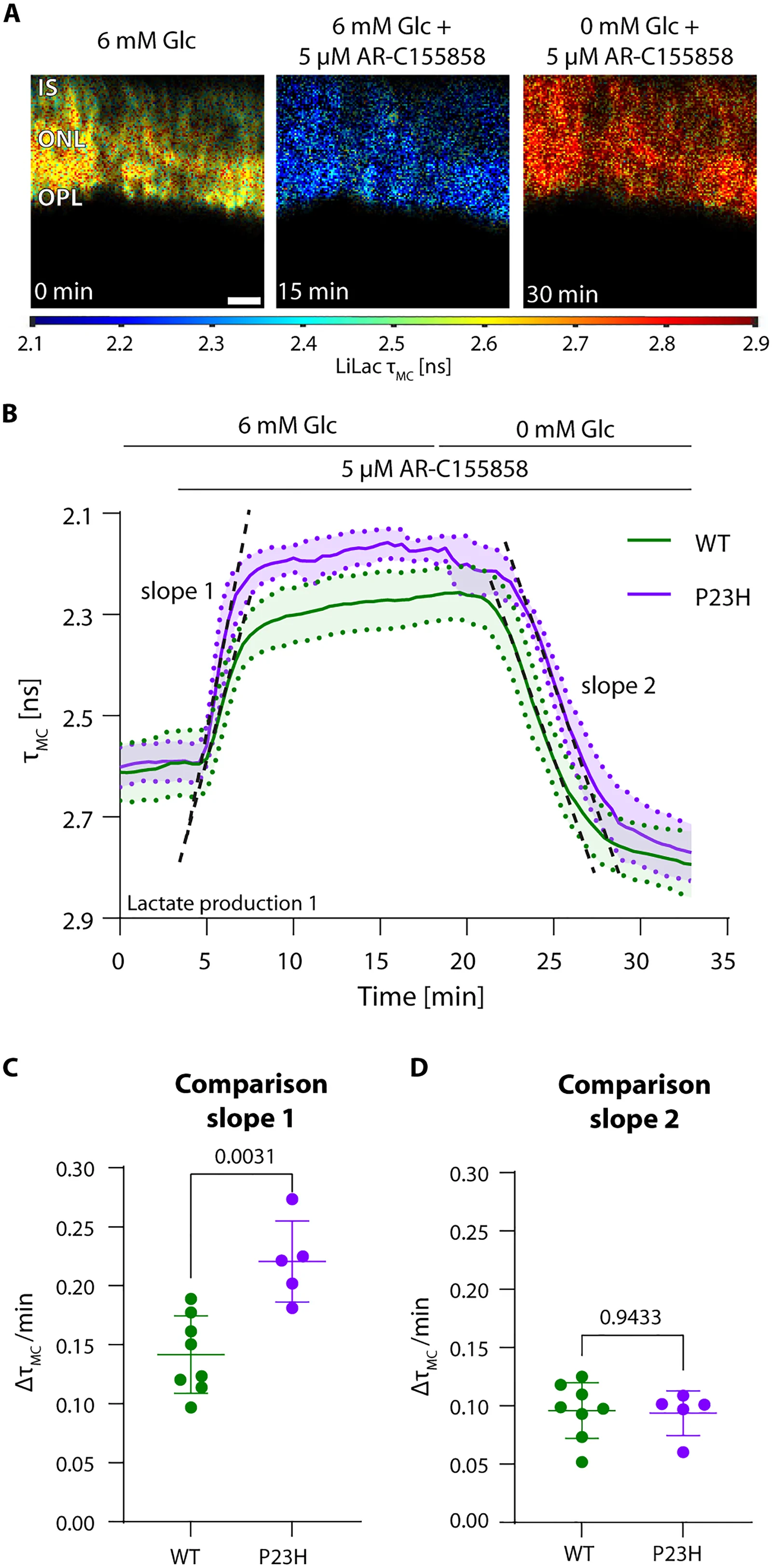
Lactate production in rods of *Rho^P23H/+^* mice. (**A**) 2P-FLIM images (τ_MC_) of the lactate sensor LiLac in rods of *Rho^P23H/+^* mice during lactate production 1 (Table 1). Scale bar: 10 μm. (**B**) Lifetime traces (means ± SD) of LiLac in rods of wt (green; data from Fig. 4D) and *Rho^P23H/+^* mice (violet; *N* = 5, *n* = 4) during lactate production 1 (Table 1). Black dashed lines: slopes used to calculate the lifetime changes of LiLac in wt and *Rho^P23H/+^* mice. (**C** and **D**) Lifetime changes (∆τ_MC_/min) in LiLac during the MCT block in the presence of glucose [(C); slope 1; lactate production] and in the absence of glucose [(D); slope 2] in rods of wt (green; data from Fig. 4, E and F) and *Rho^P23H/+^* mice (violet). Shown are individual values and means ± SD. Mann-Whitney nonparametric test. IS, inner segments; ONL, outer nuclear layer; OPL, outer plexiform layer.

## DISCUSSION

A recent study calculated that more than 50% of the ATP in the adult retina is generated through the glycolytic pathway (*60*), confirming the central role of glycolysis in retinal energy metabolism. However, this finding does not resolve which specific cell types are the main contributors. Despite differences in function, metabolic enzyme profiles and blood supply between photoreceptors and inner retinal neurons, their metabolic profiles have not been directly compared. Here, by combining 2P-FLIM of biosensor imaging with pharmacological protocols, we revealed substantial metabolic differences between rod photoreceptors and inner retinal neurons.

### OXPHOS and aerobic glycolysis

Mitochondrial respiration in rod photoreceptors is partially uncoupled from ATP production (*61*, *62*), indicating that the maximal ATP yield from glucose is not fully achieved through OXPHOS. Consequently, rods may need aerobic glycolysis to supplement ATP production. Enhanced aerobic glycolysis is consistent with their high anabolic demand, as this process maintains an essential carbon flux into lipid, amino acid, and nucleotide biosynthesis. Consistent with this metabolic organization, our data showed that inhibiting OXPHOS only partially reduced ATP levels in rods. Full depletion occurred only when glycolysis was also blocked by glucose removal. Moreover, lactate and glutamine, which support mitochondrial respiration but not glycolysis, were unable to sustain normal ATP levels. Together, these findings directly demonstrate that rod photoreceptors not only utilize but also require aerobic glycolysis alongside mitochondrial respiration to meet their energy demand. Because they engage both metabolic pathways, rod photoreceptors have a significant advantage in managing metabolic disturbances, enhancing their resilience to stress caused by environmental factors or disease. Indeed, photoreceptors without *Cox10* or *Ldha* can handle the arising metabolic imbalance for some time, but they cannot tolerate the changes in ATP production and/or anabolic processes long-term (*12*, *15*, *26*).

In contrast to rods, inner retinal neurons fully maintained baseline ATP levels in the presence of lactate. Moreover, when OXPHOS was blocked, ATP levels dropped almost completely, and the additional removal of glucose caused only a minimal further decline. This shows that inner retinal neurons mainly use OXPHOS to produce ATP and are only capable of performing aerobic glycolysis to a very limited extent, if at all.

It would be informative to estimate the relative ATP contributions of the individual pathways, the proportion of glucose converted to lactate, or the abundance of glycolytic intermediates generated. However, accurate quantification of these processes with genetically encoded sensors is currently limited due the intrinsic difficulty of calibrating metabolic sensors across distinct cellular environments (*63*, *64*).

### Alternative fuels

Our data indicate that rod photoreceptors can partially, and inner retinal neurons can fully maintain ATP levels when lactate is provided instead of glucose. This suggests that the cells, including rods, likely metabolize lactate via LDH to support OXPHOS. The five tetrameric LDH isoenzymes differ in their kinetic properties, but all catalyze the reversible interconversion of pyruvate and lactate. Because the direction of the LDH reaction depends on cellular conditions such as substrate availability and redox state, isoenzyme composition alone does not strictly determine whether a cell produces or consumes lactate (*65*, *66*). Although rod photoreceptors are enriched in LDH-5 (*15*, *19*), composed of *Ldha* encoded M subunits that preferentially support lactate production, the reaction remains reversible. Therefore, under conditions such as high extracellular lactate or limited glucose availability, rods can likely oxidize lactate to fuel OXPHOS. However, because rods rely on both glycolysis and partially uncoupled OXPHOS, lactate can only partially sustain ATP levels, as it bypasses glycolysis and fuels mitochondria less efficiently. In contrast, inner retinal neurons, which predominantly rely on OXPHOS, can maintain ATP at baseline levels using lactate alone.

As an alternative to the direct catabolism of lactate to pyruvate in photoreceptors, neighboring Müller cells could convert lactate to glutamine, which is released and taken up by rod photoreceptors. In rods, glutamine can be metabolized via glutaminolysis, where glutaminase (*67*) converts it to glutamate. This is then further converted into alpha-ketoglutarate, which enters the TCA cycle and supports OXPHOS for ATP production. Consistent with this, exogenously supplied glutamine maintained intermediate ATP levels, suggesting that rods may use glutaminolysis to direct lactate-derived glutamine into the TCA cycle and OXPHOS for ATP production. Since the available inhibitors for glutamine transport or glutaminase activity were not reliably effective in our system, future studies using genetic models will be required to determine whether rods utilize lactate directly or indirectly via conversion to glutamine by neighboring cells.

Unlike in rods, the ATP levels in inner retinal neurons declined only slowly in the absence of glucose. Either their ATP consumption was lower, or other cells supplied alternative fuels, such as lactate, for ATP production through OXPHOS, as suggested by others (*27*). Although clear evidence is missing, such a lactate shuttle in the retina is discussed between Müller glia and neurons (*68*) in analogy to the astrocyte-neuron shuttle in the brain (*69*, *70*). However, since blocking lactate transport did not accelerate the ATP depletion rate in inner retinal neurons in the absence of glucose, our data does not favor the presence of such a lactate shuttle. In addition, blocking glycogenolysis in the absence of glucose significantly increased the ATP depletion rate (fig. S5), even though lactate transport was not blocked. This additionally challenges the presence of a lactate shuttle to inner retinal neurons and instead suggests that inner retinal neurons can use glucose-6-phosphate, the breakdown product of glycogen, to produce some of their ATP through OXPHOS. Whether glycogenolysis occurs in the neurons or in adjacent Müller glia (or other) cells still needs to be determined (*71*–*74*). As neither blocking lactate transport nor inhibiting glycogen breakdown accelerated ATP depletion in inner retinal neurons to a rate similar to that observed in glucose-starved rods, our data further support the notion that rods consume energy faster than inner retinal neurons.

We need to emphasize that by expressing the biosensors under the control of the human synapsin 1 (hSyn1) promoter fragment (*75*), we analyzed metabolites in a heterogeneous cell population of ganglion and amacrine cells in the inner retina (fig. S1C). Thus, we cannot specify the individual metabolic properties of these cells, nor can we determine the contribution of the various cellular compartments, which may differ in their mitochondrial content and distribution as well as local energy metabolism (*16*, *76*).

### Metabolism in degenerating rods of the *Rho*^*P23H/+*^ mouse

Neurodegeneration is often accompanied by critical mitochondrial dysfunction and stress even before onset of the disease (*77*) and by the formation of reactive oxygen and nitrogen species (*78*). The low mitochondrial reserve capacity of rods (*79*) may make these cells especially susceptible to stress caused by environmental conditions or gene mutations. To study the effects of a mutation that causes photoreceptor degeneration in RP patients, we investigated the ATP and lactate dynamics in rod photoreceptors of the *Rho^P23H/+^* mouse (*59*) during degeneration. Our data revealed that ATP levels declined faster in the degenerating rods when lactate substituted for glucose (Fig. 5F). Since the ATP depletion rates were comparable when no fuel was present, the ATP usage was similar in *Rho^P23H/+^* and wildtype rods, but the production through OXPHOS was slowed in the disease model. This points to impaired mitochondrial function, a common feature in neurodegenerative diseases (*78*).

Reduced mitochondrial function may increase glycolysis and LDH activity as a compensatory response (*80*, *81*) resulting in heightened lactate production, as detected when lactate export was blocked in the presence of glucose (Fig. 6B). Although this additional lactate may be released from the cells in vivo, it likely does not fully compensate for loss of lactate production from degenerated rods. According to the proposed metabolic landscape in the outer retina, this would leave less lactate available to the RPE cells, causing them to rely more on glucose. As a result, glucose availability for the remaining photoreceptors would be restricted, potentially further impairing their metabolism (*82*, *83*). Since our experimental setup, which contains abundant metabolites in the medium during 2P-FLIM measurements, cannot mimic the in vivo situation with regulated glucose transport through the RPE, such changes may not be detectable with this approach (see limitations below). In addition, the slow disease progression in the *Rho^P23H/+^* mouse (*59*) resulted in the presence of a heterogeneous population of rods at different stages of degeneration. This may have averaged potentially divergent metabolic profiles in rods during early and late phases of degeneration and may explain why we detected only relatively mild changes in this model. It is important to note, however, that the mutant rods retained their capability to efficiently metabolize glucose, suggesting that supporting energy metabolism might be a promising therapeutic strategy to improve mitochondrial function in disease, as has been suggested previously (*84*–*88*).

### Limitations, conclusions, and future directions

A limitation of our study is the use of acute eye sections which results in the unrestricted access of the cells to the metabolites in the medium. This differs from the in vivo situation, where the blood/retina barrier, the transport of glucose through the RPE, and the distance from the supplying blood vessels define not only the metabolite abundance and composition, but also generate a concentration gradient for the nutrients in the tissue (*5*, *89*). Nevertheless, the versatility of genetically encoded metabolic biosensors, combined with 2P-FLIM and pharmacological protocols, enabled the dissection of the metabolism on a cellular level. While we demonstrate that rods can use glutamine and can likely metabolize lactate, we show that these alternative fuels are not sufficient to produce basal ATP levels. Our data further show that blocking OXPHOS does not fully deplete rods of ATP. Taken together, we conclude that rod photoreceptors perform aerobic glycolysis but simultaneously need OXPHOS to maintain their ATP levels. In contrast, inner retinal neurons depend almost exclusively on OXPHOS and can efficiently metabolize lactate.

It will be of strong interest to compare the rod data to the metabolism in cones in the future, as their higher need for ATP (*90*) may increase relative contribution of OXPHOS to total ATP production. Although we cannot determine the metabolic dynamics in dark-adapted wildtype rods because two-photon imaging causes rods to enter a light-activated state, it may be possible to do so in cones with their reduced light sensitivity. Such data can then be compared to data from mice lacking transducin (*Gnat1^−/−^*) that mimic the dark situation because phototransduction cannot be activated. Lastly, the suggested shift from OXPHOS to glycolysis in degenerating rods requires further experiments investigating energy metabolism at different stages of degeneration and across additional disease models, including models for cone-rod dystrophies.

## MATERIALS AND METHODS

### Study design

The objective of this study was to dissect the metabolic differences between rod photoreceptors and inner retinal neurons, as well as to determine whether rod photoreceptor metabolism is impaired in the *Rho^P23H/+^* mouse, a model of RP. To achieve this, we expressed genetically encoded biosensors under the control of cell type-specific promoters using AAV-mediated gene transfer to the retinal neurons. To determine the intracellular levels of ATP and lactate, as well as the redox status of the cells, and to resolve the metabolic dynamics of the different neurons, we applied a variety of pharmacological protocols and measured the fluorescence lifetime of the biosensors using 2P-FLIM. All animal experiments and animal care were conducted in accordance with institutional regulations of the University of Zürich and the regulations of the veterinary authorities of the Kanton of Zürich.

### Animals

All animal experiments were approved by the Cantonal Veterinary Office of Zurich, Switzerland (license numbers: ZH019/2019, ZH091/2019, ZH105/2022, ZH065/2025) and adhered to the ARVO statement for the use of Animals in Ophthalmic and Vision Research. Wildtype (wt) mice (129S6, C57B6/J) and transgenic mice (*Rho^P23H/+^*) (*59*) on a C57B6/J background were maintained as breeding colonies at the Laboratory Animal Services Center (LASC) of the University of Zurich with a 14/10 h light-dark cycle with an average light intensity of 60–150 lux at cage level. BALB/c mice were obtained from Charles River (Hilden, Germany). Mice had access to food and water ad libitum.

### Cloning and virus production

The cDNAs encoding the biosensors for calcium [GCaMP6s (*91*)], ATP [ATeam1.03 (*42*), (Viral Vector Facility of the Neuroscience Center Zurich; VVF)], lactate [LiLac (*55*) (Addgene #184570)] and NADH/NAD^+^ [Peredox (*52*); VVF] were cloned behind the rod-specific mOP promoter (*26*, *33*) and the hSyn1 promoter (*75*). To avoid recombination during AAV production, a codon-diversified version of ATeam1.03 was used (*92*). The construct containing GCaMP6s was packaged into the AAV8(double Y-F + TV)/2 capsid (*26*, *93*). Constructs containing ATeam1.03, LiLac and Peredox were packaged into the AAV2(QuadYF+TV; 7 m8)/2 capsid (*94*) for transduction of rods after subretinal injection, or the AAV6 capsid for transduction of inner retinal neurons after intravitreal injection. To label RPE cells the mCherry cDNA was cloned behind the VMD2 promoter (*32*) and packaged into the AAV4 capsid. All AAVs were produced by the VVF of the Neuroscience Center Zurich.

### Intraocular injection of AAVs and funduscopy

Intraocular injections were performed as described previously (*95*). Briefly, prior to injection pupils of the mice were dilated under dim light with 1 drop of 1% Cyclogyl (Alcon Pharmaceuticals, Fribourg, Switzerland) and 1 drop of 5% Neosynephrin (Ursapharm Schweiz GmbH, Hünenberg, Switzerland). The mice were anesthetized under dim light with a single subcutaneous injection of Ketamine (85 mg/kg, Pfizer AG, Zürich, Switzerland) / Xylazine (10 mg/kg, Elanco Animal Health GmbH, Basel, Switzerland) using a syringe with a 30G needle. Lacrinorm Carbomerum (Bausch & Laumb Swiss AG, Zug, Switzerland) was applied to keep the eyes moist. Anesthetized mice were placed on a heating pad set at 37°C and the head was fixed in a stereotactic adapter (Hugo Sachs Elektronik – Harvard Apparatus GmbH, March-Hugstetten, Germany). The temporal sclera was punctured with a 30G needle just below the ora serrata. A 34G blunt end needle mounted on a 5 μl Hamilton syringe (Hamilton Bonaduz AG, Bonaduz, Switzerland), containing the AAV solution, was carefully inserted into the vitreous or the subretinal space through the puncture site using a micromanipulator (H. Saur Laborbedarf, Reutlingen, Germany). A cover slip was placed on top of the Lacrinorm gel for better visibility of the needle. Rod photoreceptors were transduced by a transvitreal subretinal injection of 1 μl AAV solution (1 × 10^9^ vg/μl) nasally to the optic nerve. Inner retinal neurons were transduced by an intravitreal injection of 1-4 × 10^9^ vg/μl AAVs (in 1 μl) above the optic nerve head. AAV solutions contained 10% fluorescein (0.1 mg/ml, Akorn Inc., IL, USA) to visually control the injections. After the procedure, the anesthesia was reversed by the subcutaneous injection of Atipamezol (1.67 mg/kg, Graeub, Bern, Switzerland) and mice were placed on a heating pad until fully awake. Wildtype mice were injected at 4–6 weeks of age and *Rho^P23H/+^* mice were injected at 4 weeks of age.

One to three weeks after injection, mice were subjected to fluorescent funduscopy and optical coherence tomography (OCT) using the Micron IV system (Phoenix Research Labs, Pleasanton, CA, USA) as described (*96*). Anesthesia and pupil dilation were performed as described above. Eyes with no transgene expression, injection-inflicted retinal damage or bleeding were excluded from the study.

### Immunofluorescence

Immunofluorescence was performed as previously described (*26*). Briefly, mice were euthanized two weeks after injections, and eyes were marked nasally, enucleated and fixed for 1.5 hours in 4% paraformaldehyde (PFA) in PBS at 4°C. The cornea and lens were removed, and the eyes were fixed for another 2.5 hours in PFA, followed by cryoprotection in 30% sucrose for 2 hours, embedding and freezing in O.C.T. medium (O.C.T., 81–0771-00, Biosystems Switzerland AG, Nussloch, Germany). A Leica cryostat (Biosystems Switzerland AG, CM1860) was used to prepare 12-μm-thick sections. Cryo-sections were blocked using PBS containing 3% normal goat serum (Sigma Aldrich, G9023, St. Louis, MO, USA) and 0.3% Triton X-100 (Sigma Aldrich, T9284) followed by incubation with the following primary antibodies overnight at 4°C: rabbit anti-ARR3 (1:1000; Merck Millipore Chemicals, Darmstadt, Germany; ab15282), mouse anti-CALB2 (1:1000; Merck Millipore Chemicals; MAB1568) and rabbit anti-RBPMS (1:200; Phosphosolutions, Aurora, CO, USA; 1830-RBPMS). After washing with PBS, the secondary antibodies (goat anti-rabbit IgG Alexa Fluor 568, A11011; Anti-mouse IgG Alexa Fluor 568, A11004; ThermoFisher (Waltham, MA, USA); 1:500) were applied for 1 hour at room temperature. Mowiol (Mowiol 4–88, 475904, Calbiochem, Darmstadt, Germany) antifade medium was used to mount the sections. Fluorescence images were acquired with a confocal microscope (Andor BC43 CF, Oxford instruments, Abingdon, UK). The images were processed using ImageJ (Version 1.54f, National Institutes of Health).

### Morphology

For morphological analysis, eyes were fixed in 2.5% glutaraldehyde in 0.1 M cacodylate buffer, pH 7.2, at 4°C overnight. For each eye the posterior and inferior half were prepared, post-fixed in 1% osmium tetroxide in 0.1 M cacodylate buffer and embedded in EPON 812 (Biolyst Scientific, Morgantown, PA, USA) as described (*97*). Sections (500 nm) were cut through the optic nerve head, stained with toluidine blue and imaged by light microscopy (AxioImager Z2, Carl Zeiss AG, Feldbach, Switzerland).

### Acute eye section preparations for ex vivo imaging

The mice were housed in a room with a reversed 12/12 hours light/dark cycle for at least 10 days before the experiments. Acute eye sections of wildtype mice were prepared at 8–12 weeks of age and of *Rho^P23H/+^* mice at 8 weeks of age, which was 4–5 weeks (wildtype) or 4 weeks (*Rho^P23H/+^* mice) after injections. Mice were euthanized at 10 a.m. during the subjective night. Eyes were marked nasally and embedded in 3% agarose (Type III-A, Sigma, A9793) dissolved in freshly prepared artificial cerebrospinal fluid (ACSF) containing: 126 mM NaCl, 3 mM KCl, 2 mM CaCl_2_, 1.25 mM NaH_2_PO_4_, 26 mM NaHCO_3_, 2 mM MgSO_4_, 6 mM D-glucose. All ACSF used for tissue sectioning, incubation, and imaging was continuously bubbled with oxycarbon (95% O_2_/5% CO_2_, 800001855, Linde Gas Schweiz, Dagmersellen, Switzerland). Eyes were cut temporal to nasal into 300-μm-thick acute sections using a microtome with a horizontally oscillating blade (Microm HM 650 V, Histocom AG, Zug, Switzerland) using the following settings: feed = 300, trim = 25, frequency = 85, amplitude = 1, speed = 7. The sections were cut and collected in ice-cold ACSF, then transferred to a petri dish containing ACSF for equilibration at room temperature. After 2 hours, the first section was transferred to an imaging chamber containing ACSF, which was slowly warmed to an imaging temperature of 35°C for 10–15 min. The last section was transferred and imaged no later than 4 hours after the first section. While the duration in the equilibration medium did not evidently impact the stability of the fluorescence or lifetime signals of the sensors, we ensured that we imaged the sections after various equilibration times. The section was held in position by a slice anchor (SS SLIC HD F/RC-26 CHAM 1.5 mm, 640251, MultiChannel Systems HEKA, Holliston, USA) to prevent movement during imaging. The imaging chamber (approximate volume: 2.5 ml) was perfused with continuously oxygenated ACSF at a flow rate of approximately 1.7 ml/min and the temperature was maintained at 35°C using a temperature controller (V TC05, Luigs & Neumann GmbH, Ratingen, Germany). Imaging was performed at the depth of 30–80 μm below the section surface. For experiments using ACSF with 0 mM D-glucose concentration, glucose was substituted with sucrose.

### Two-photon excitation laser scanning microscopy (2P) and two-photon fluorescence lifetime microscopy (2P-FLIM)

A custom-made two-photon laser scanning microscope (*98*) equipped with a tunable femtosecond pulsed laser (Chameleon Discovery NX with Total Power Control, Coherent, Saxonburg, PA, USA) and a 20x water immersion objective (W Plan-Apochromat 20x/1.0, Zeiss) was used for image acquisition during two-photon excitation laser scanning microscopy (2P) and two-photon fluorescence lifetime microscopy (2P-FLIM). Excitation and emission beam paths were separated by a dichroic mirror (KS93 Cold Light Mirror, 685 nm LP, QiOptiq, Rhyl, UK). Dichroic mirrors at 506 nm (F38–506; AHF Analysentechnik, Tübingen, Germany) and 560 nm (F38–560; AHF Analysentechnik) further separated the emission light into colored components, that were then focused on GaAsP photomultipliers (H10770PA-40sel; Hamamatsu Photonics, Hamamatsu, Japan) equipped with filters for cyan (Brightline HC 475/50; AHF Analysentechnik), green/yellow (Brightline HC 542/50; AHF Analysentechnik) and red (Brightline HC 607/70; AHF Analysentechnik) wavelengths. ScanImage 3.8 (*99*) and a custom-written LabVIEW software (Version 2012; National Instruments) were used for image control and data acquisition. For FLIM acquisition in a given channel, the GaAsP photomultiplier was replaced with a hybrid photomultiplier detector (PMA Hybrid 40 mod, PicoQuant, Berlin, Germany), and the signal collected and processed via a custom designed FLIM acquisition hardware and software based on an FPGA evaluation board (*100*). For FLIM in the cyan channel, an additional Brightline HC 475/50 filter was employed to eliminate any residual contribution from second harmonics generation.

### 2P Calcium imaging and analysis

Acute eye sections expressing GCaMP6s in rod photoreceptors and mCherry in RPE cells were co-excited with 940 nm and 1040 nm and emitted fluorescence intensity was acquired at a 0.74 Hz frame rate and 512 × 512 pixel resolution.

To measure Ca^2+^ levels in rod photoreceptors, GCaMP6s was excited at 940 nm with a laser power of 8–23 mW. Emitted fluorescence intensity was acquired at a 5.92-Hz frame rate and 128 × 128 pixel resolution. Rod photoreceptors were imaged for 10 s with 30 s recovery under continuous ACSF flow. To evaluate the contribution of calcium influx through cGMP-gated channels in photoreceptor cells, 2 mM 8-pCPT-cGMP (table S1) was added to the ACSF and rods were continuously imaged for 200 s. To assess the impact of switching from glucose to lactate on Ca^2+^ levels, rod photoreceptors were imaged for 10 s with 30 s recovery period under continuous ACSF flow, in accordance with the "lactate recovery" protocol (Table 1). For data analysis regions of interest (ROI) were manually outlined around photoreceptor segments, including inner and outer segments, using ImageJ. Further data analysis was conducted using a custom-made MATLAB (MathWorks, R2018b) script. The Ca^2+^ traces (ΔF/F) were calculated from each ROI using the first frame of each experiment as baseline.

### 2P-FLIM of metabolic biosensors for ATP, lactate, and NADH/NAD^+^

Imaging of the metabolic biosensors was performed using various pharmacological protocols (Table 1) at 2.96-Hz frame rate and 128 × 128 pixel resolution. The fluorescence lifetime of the sensors was collected at intervals of 25 s. 30 images (for the acquisition of fluorescence lifetime of LiLac and Peredox) or 20 images (for ATeam1.03) were averaged within the 25-s time interval. ATeam 1.03 and LiLac were excited at 870 nm using 3–12 mW laser power. Peredox was excited at 800 nm wavelength using 5–13 mW laser power. Detailed information about imaging protocols and drugs are listed in Table 1 and table S1, respectively.

### 2P-FLIM analysis

FLIM images were analyzed using custom-made MATLAB (MathWorks, R2020a) scripts based on the FLIMfit 5.1.1 library (*101*) using biexponential fitting and the method-of-moments (MOM), as previously described (*100*). Although all analyses were broadly consistent, we noticed the presence of very-short lived autofluorescence, especially in dimmer areas corresponding to the photoreceptor outer segments. To reduce its impact, regions-of-interests excluding outer segments were selected, and the average fluorescence decays in this region was analyzed. To analyze fluorescence lifetime changes in the inner retina, the average fluorescence decay of the whole frame was analyzed.

To further minimize the contribution of short-lived contaminations, we first detected the center-of-mass of the IRF (Instrument Response Function), and aligned the decays such that it would fall 250 ps from the start of the time-scale. We then excluded the first 750 ps of the signal (corresponding to roughly 500 ps after the peak of excitation), as they would be the most affected by the contaminating signal. Finally, we conducted a MOM analysis on the cropped decays (“MOM cropped”, which we will refer to as τ_MC_).

The slopes were calculated using the linear function: *y* = *mx* + *b* (where *m* represents the slope) in GraphPad Prism version 10.5.0 (GraphPad Software, San Diego, CA, USA).

### Statistical analysis and presentation

The numbers of mice (*n*) and slices (*N*) are detailed in the figure legends. GraphPad Prism Version 10.5.0 was used for data visualization, and the Mann-Whitney nonparametric test was used to compare data.
