# Supplementary material for: Rod photoreceptors have a dual dependency on both aerobic glycolysis and OXPHOS and diverge metabolically from other retinal neurons
Source: Sci Adv. 2026 Oct 7;12(41):eaeg8687. doi: 10.1126/sciadv.aeg8687 (PMC13644791; doi:10.1126/sciadv.aeg8687)
Supplement: Supplementary file 1 [file sciadv.aeg8687_sm.pdf]

Science Advances
AAAS

Supplementary Materials for

Rod photoreceptors have a dual dependency on both aerobic glycolysis and OXPHOS and diverge metabolically from other retinal neurons

Gabriele M. Wögenstein *et al.*

Corresponding author: Christian Grimm, cgrimm@opht.uzh.ch

Sci. Adv. **12**, eaeg8687 (2026)
DOI: 10.1126/sciadv.aeg8687

This PDF file includes:

Figs. S1 to S8
Table S1

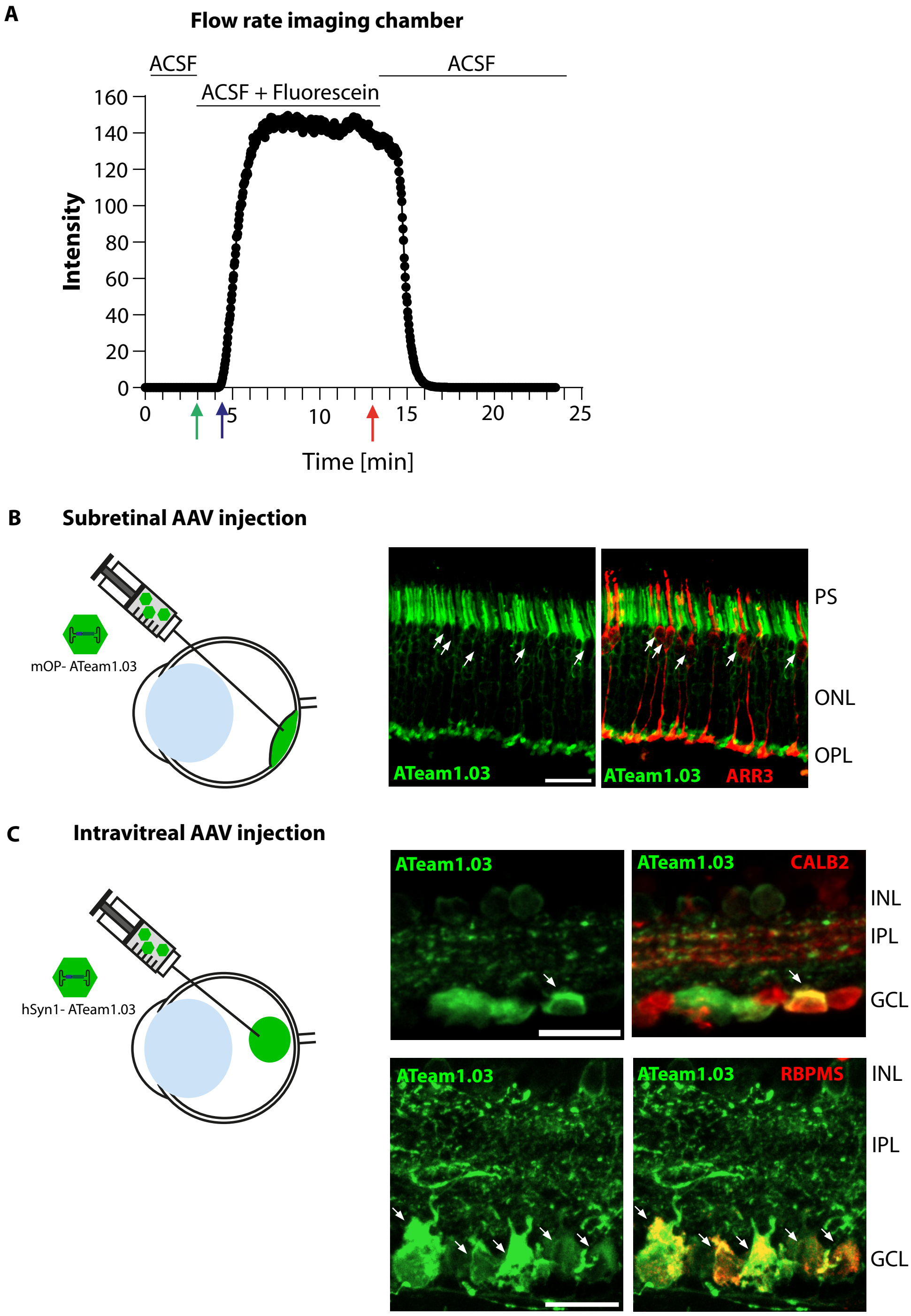

Fig. S1: The flow-rate in the imaging chamber and the cell-type specificity of biosensor expression. A, Representative intensity trace of fluorescein. Baseline fluorescence intensity during inflow of ACSF was recorded for 3 min, followed by inflow of fluorescein ($1x10^{-5}$ M fluorescein in ACSF; green arrow: start of inflow) for 10 min. Fluorescein arrived in the chamber after approx. 1.4 min of inflow (blue arrow). After 10 min, fluorescein was washed out with ACSF (red arrow: start of washout). **B,** Subretinal delivery of AAV::mOP-ATeam1.03 (left) and retinal cross-sections showing ATeam1.03 fluorescence (green) and cone arrestin immunofluorescence (right, ARR3, red). White arrows indicate ARR3-positive but ATeam1.03-negative cells. **C,** Intravitreal delivery of AAV::hSyn1-ATeam1.03 (left) and cross-sections showing ATeam1.03 expression (green) and calretinin (CALB2, red, right upper panel) or RNA binding protein with multiple splicing (RBPMS, red, right lower panel) immunofluorescence. White arrows indicate cells expressing ATeam1.03 and the amacrine cell marker CALB2 or the ganglion cell marker RBPMS. Scale bars: 20 µm. GCL: ganglion cell layer. INL: inner nuclear layer. IPL: inner plexiform layer. ONL: outer nuclear layer. OPL: outer plexiform layer. PS: photoreceptor segments.

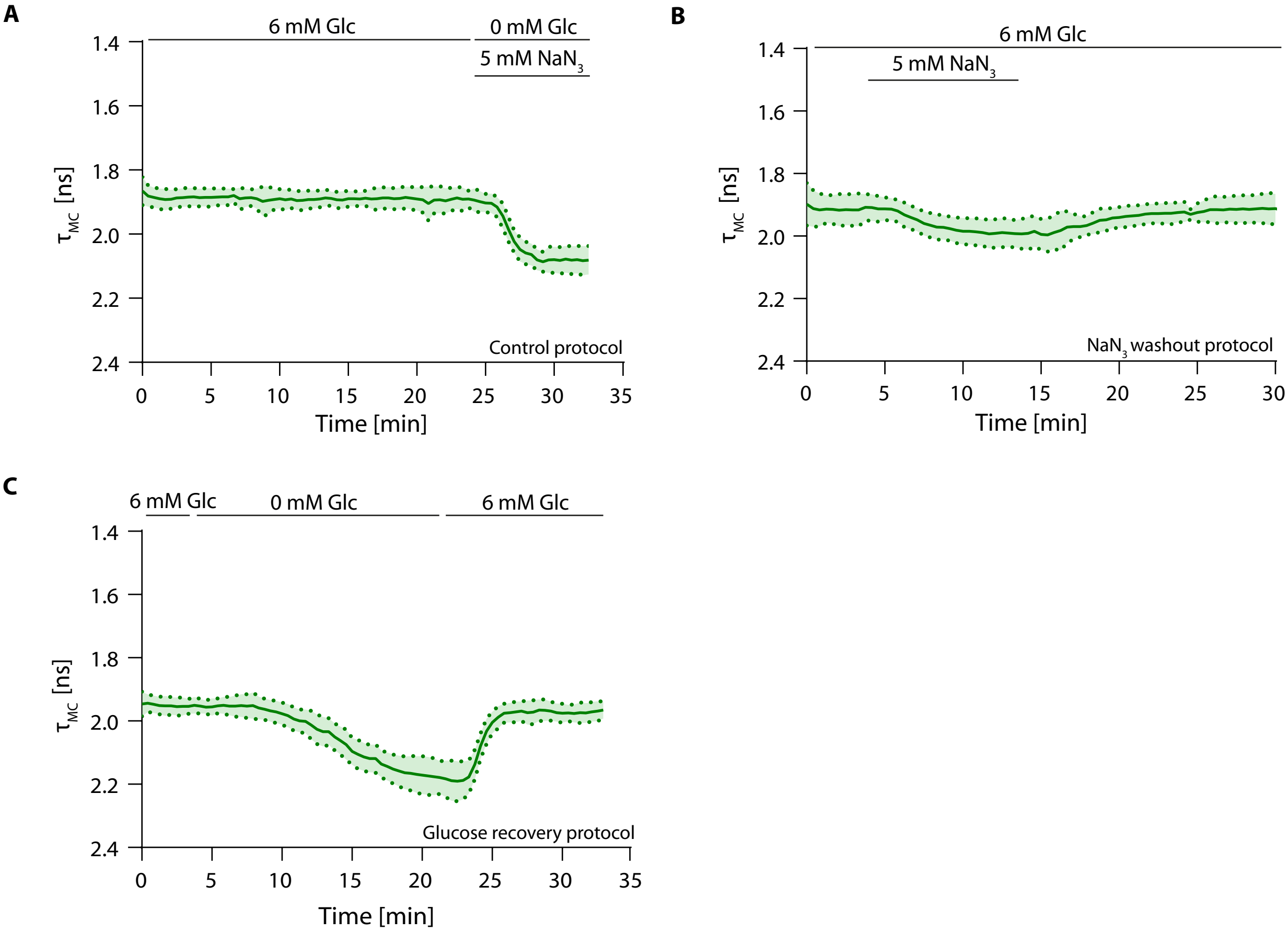

Fig. S2: Stability of ATeam1.03 fluorescence lifetime and recovery after oxidative phosphorylation block or aglycemia in rods of wildtype mice (129S6). A, Lifetime trace (mean ± SD; N = 3, n = 3) of ATeam1.03 in rods during the 'control' protocol (Table 1). **B,** Lifetime trace (mean ± SD; N = 3, n = 3) of ATeam1.03 in rods during 'NaN_3 washout' (Table 1). **C,** Lifetime trace (mean ± SD; N = 5, n = 2) of ATeam1.03 in rods during 'glucose recovery' (Table 1).

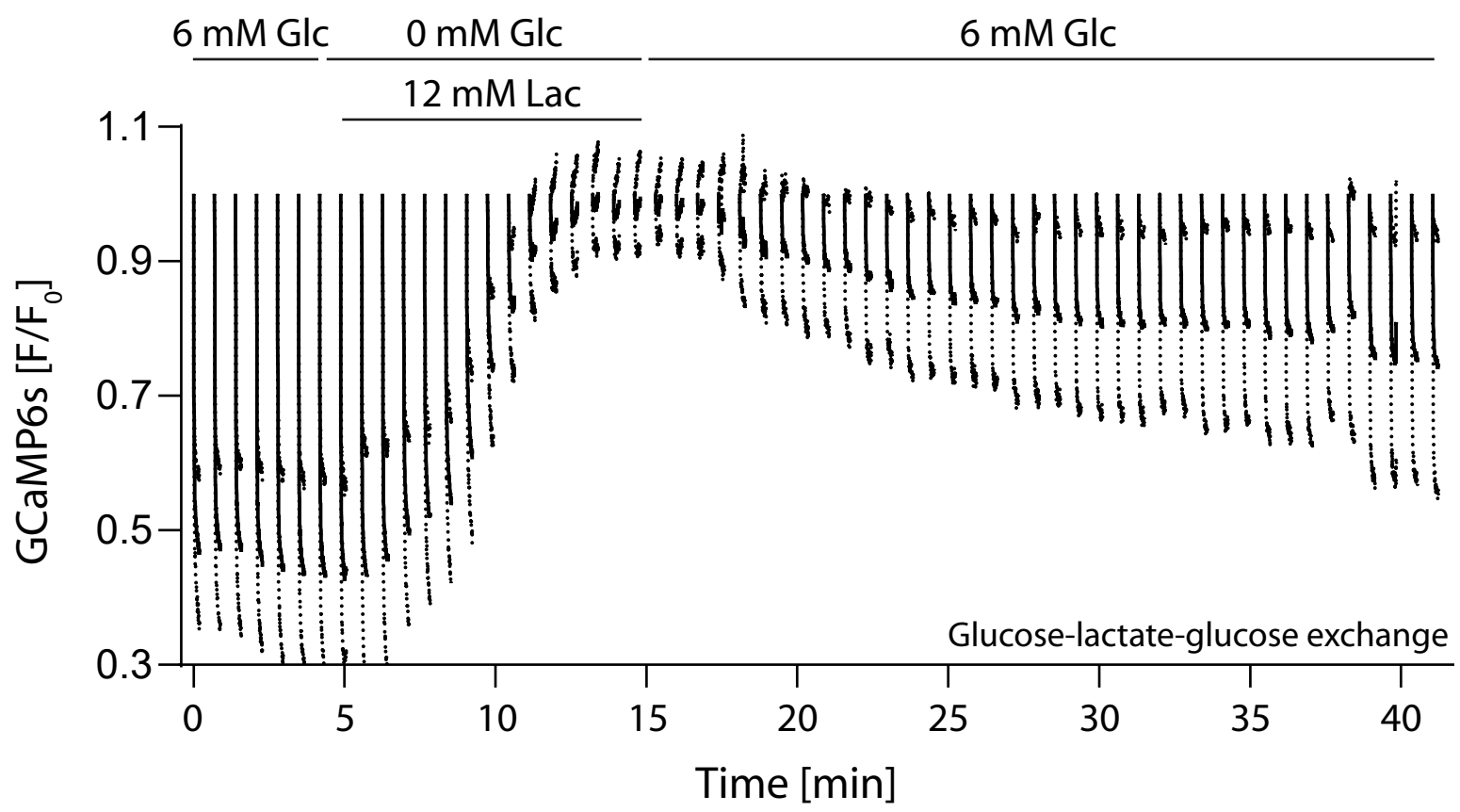

Fig. S3: Light-induced calcium response in rods of wildtype mice (129S6) during glucose-lactate-glucose exchange. GCaMP6s traces (mean ± SD; N = 3, n = 2) repetitively imaged in photoreceptor segments of wildtype mice during 10 s of 2P imaging followed by 30 s recovery during the 'glucose-lactate-glucose exchange' protocol (Table 1).

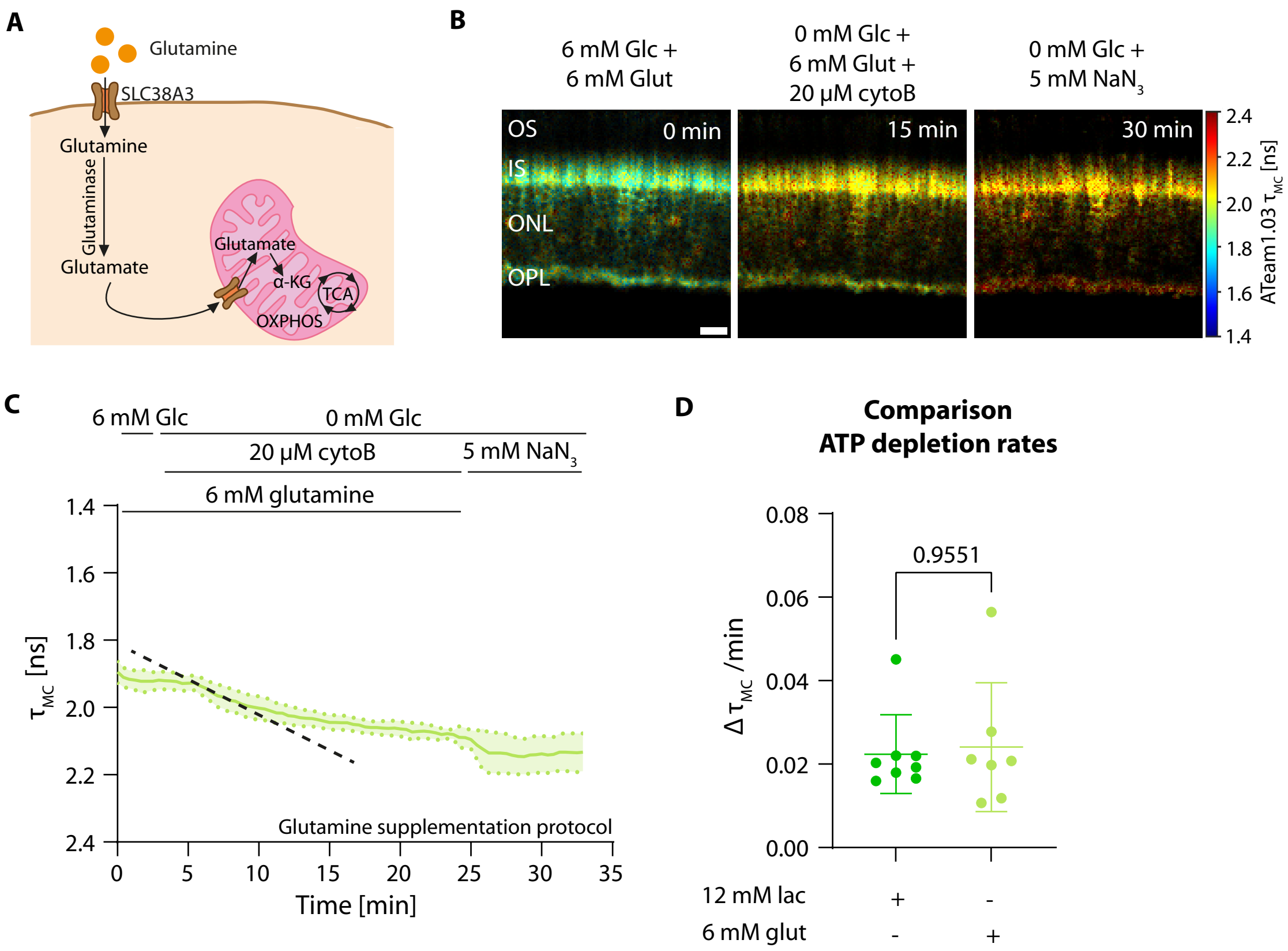

Fig. S4: ATP dynamics in rods supplied with glutamine of wildtype mice (129S6). A, Schematic representation of glutamine metabolism. **B,** 2P-FLIM images (τ_{MC}) of ATeam1.03 in rods at different time points during 'glutamine supplementation'. Scale bar: 10 µm. **C,** Lifetime traces of ATeam1.03 in rods (N = 7, n = 3) during 'glutamine supplementation' (mean ± SD). *Black dashed line:* slope used to calculate ATP depletion rates. **D,** Comparison of the ATP depletion rates ($\Delta\tau_{MC}$/min) during 'lactate supplementation' (data from Fig. 2D(c), green) and 'glutamine supplementation' (light green) in rods. Shown are individual values and means ± SD. Mann-Whitney nonparametric test. OS: outer segments. IS: inner segments. ONL: outer nuclear layer. OPL: outer plexiform layer.

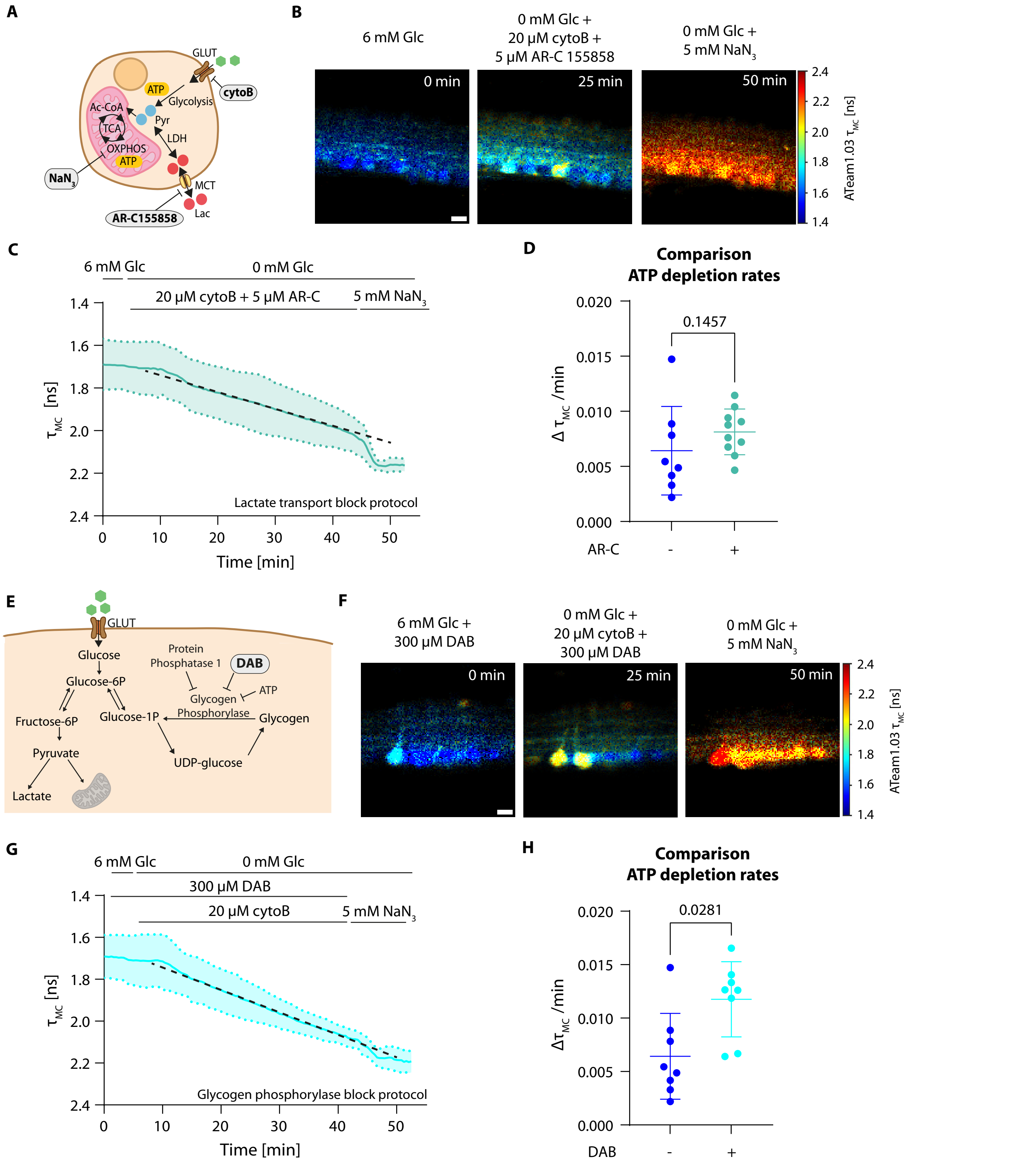

Fig. S5: Alternative fuels for ATP production in inner retinal neurons of wildtype mice (129S6). A, Schematic representation of cellular metabolism. Monocarboxylate transporters (MCT) were blocked with AR-C155858 (AR-C), glucose (Glc) uptake with cytoB and OXPHOS with NaN_3. **B,** 2P-FLIM images (τ_{MC}) of ATeam1.03 in inner retinal neurons at different time points during 'lactate transport block' (Table 1). Scale bar: 10 µm. **C,** Lifetime trace of ATeam1.03 in inner retinal neurons during lactate transport block (mean ± SD; N = 10, n = 5). *Black dashed line:* slope used to calculate ATP depletion rates. **D,** Comparison of ATP depletion rates ($\Delta\tau_{MC}$/min) during 'glucose depletion' (data from Fig. 2E(c), dark blue) and 'lactate transport block' (turquoise). Shown are individual values and means ± SD. Mann-Whitney nonparametric test. **E,** Schematic representation of glycogen metabolism and the inhibition of glycogen phosphorylase with 1,4-Dideoxy-1,4-imino-D-arabinitol hydrochloride (DAB) to prevent the breakdown of glycogen to enter glycolysis (Table 1). **F,** 2P-FLIM images (τ_{MC}) of ATeam1.03 in inner retinal neurons at different time points during 'glycogen phosphorylase block' (Table 1). Scale bar: 10 µm. **G,** Lifetime trace of ATeam1.03 in inner retinal neurons during 'glycogen phosphorylase block' (mean ± SD; N = 8, n = 5). *Black dashed line:* slope used to calculate ATP depletion rates. **H,** Comparison of ATP depletion rates ($\Delta\tau_{MC}$/min) during 'glucose depletion' (data from Fig. 2E(c), dark blue) and the 'glycogen phosphorylase block' (light blue). Shown are individual values and means ± SD. Mann-Whitney nonparametric test.

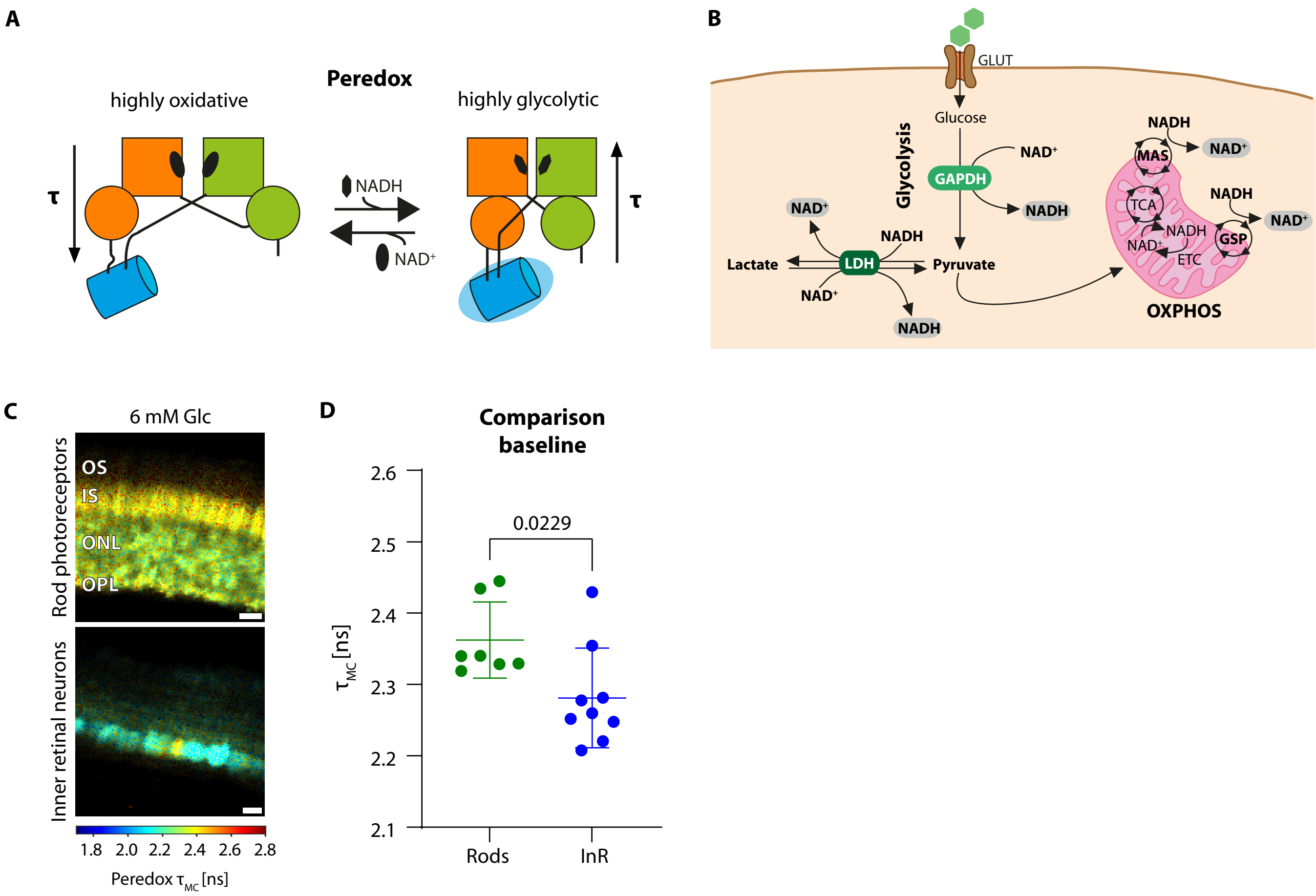

Fig. S6: Redox state measurements in rods and neurons of the inner retina of wildtype mice (129S6). A, Schematic representation of the NADH/NAD$^+$ sensor Peredox (54). The lifetime (τ) of Peredox increases when the NADH/NAD$^+$ ratio is high (glycolytic) and decreases when the NADH/NAD$^+$ ratio is low (oxidative). **B,** Schematic representation of main mechanisms influencing the cytosolic NADH/NAD$^+$ ratio in cells. **C,** 2P-FLIM images (τ_{MC}) of Peredox in rods (upper panel) and inner retinal neurons (lower panel) at baseline. Scale bars: 10 µm. **D,** Comparison of the lifetime (τ_{MC}) of Peredox at baseline levels in 6 mM glucose in rods (green) and inner retinal neurons (blue). Shown are individual values and means ± SD. Statistics: Mann-Whitney nonparametric test. OS: outer segments. IS: inner segments. ONL: outer nuclear layer. OPL: outer plexiform layer.

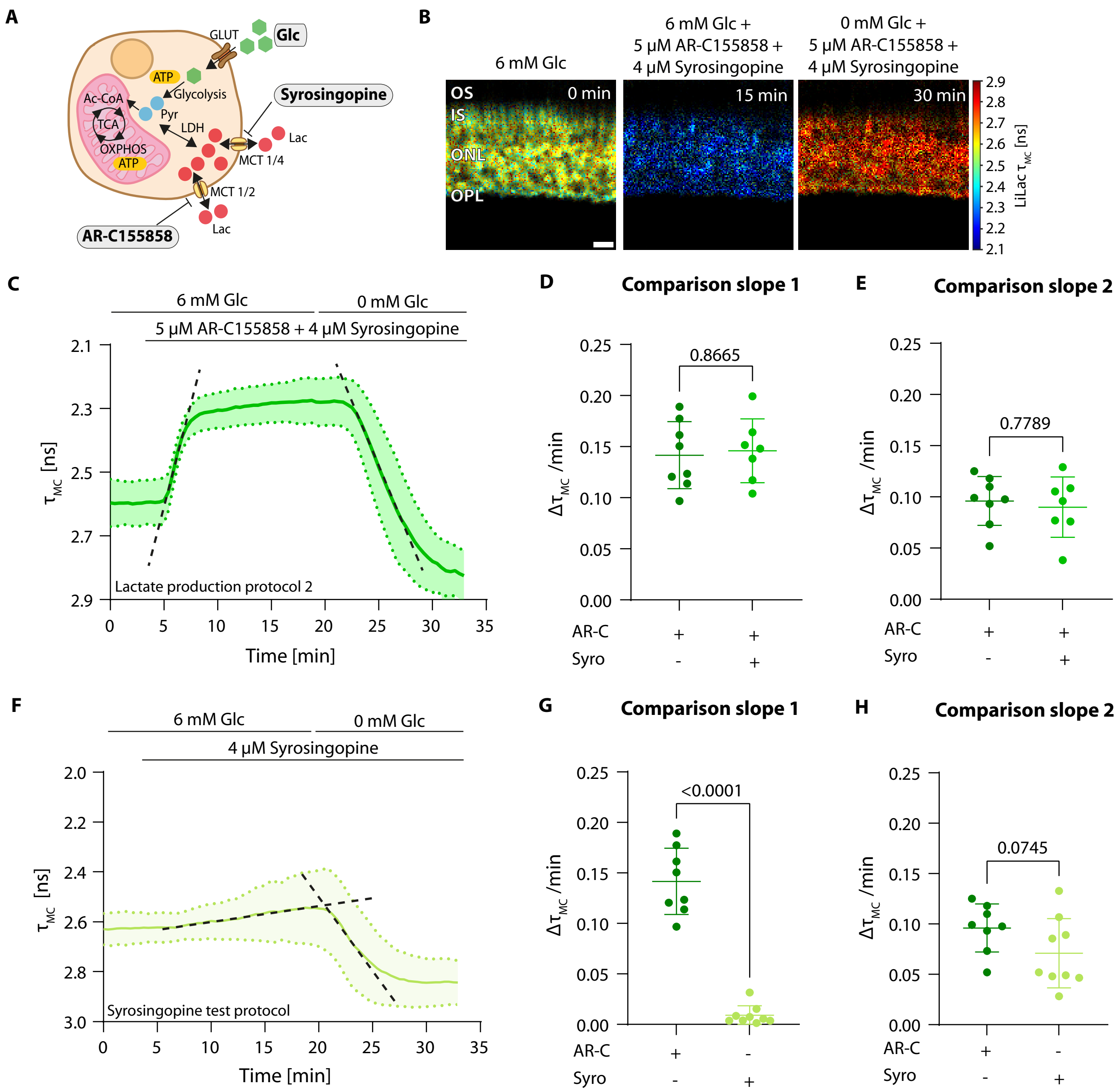

Fig. S7: Lactate accumulation by blocking different MCTs in rods of wildtype mice (129S6). A, Schematic representation of cellular metabolism, illustrating lactate transport via MCTs and their blocking with AR-C155858 (MCT1/2) and syrosingopine (MCT1/MCT4). **B,** 2P-FLIM images (τ_{MC}) of LiLac in rods at different time points during 'lactate production 2' (Table 1). Scale bar: 10 µm. **C,** Lifetime traces (means ± SD) of LiLac in rods (N = 7, n = 3) during 'lactate production 2'. *Black dashed lines*: slope used to calculate the changes in the lifetime in LiLac shown in panels D, E. **D, E,** Lifetime changes ($\Delta\tau_{MC}$/min) in LiLac during 'lactate production 1' while blocking MCT1/2 (data from Fig. 4 E, F, dark green) and during 'lactate production 2' while blocking MCT1/2 and MCT4 (bright green), in the presence of glucose (D; slope 1; lactate production) and in the absence of glucose (E; slope 2). **F,** Lifetime traces (means ± SD) of LiLac in rods (N = 9, n = 4) during the 'syrosingopine test' (Table 1). *Black dashed lines*: slope used to calculate the changes in the lifetime in LiLac shown in panels G, H. **G, H,** Lifetime changes ($\Delta\tau_{MC}$/min) in LiLac during 'lactate production 1' while blocking MCT1/2 (data from Fig. 4 E, F, green) and during 'syrosingopine test' while blocking MCT1/4 (light green), in the presence of glucose (G; slope 1; lactate production) and in the absence of glucose (H; slope 2). Shown are individual values and means ± SD. Mann-Whitney nonparametric test. OS: outer segments. IS: inner segments. ONL: outer nuclear layer. OPL: outer plexiform layer.

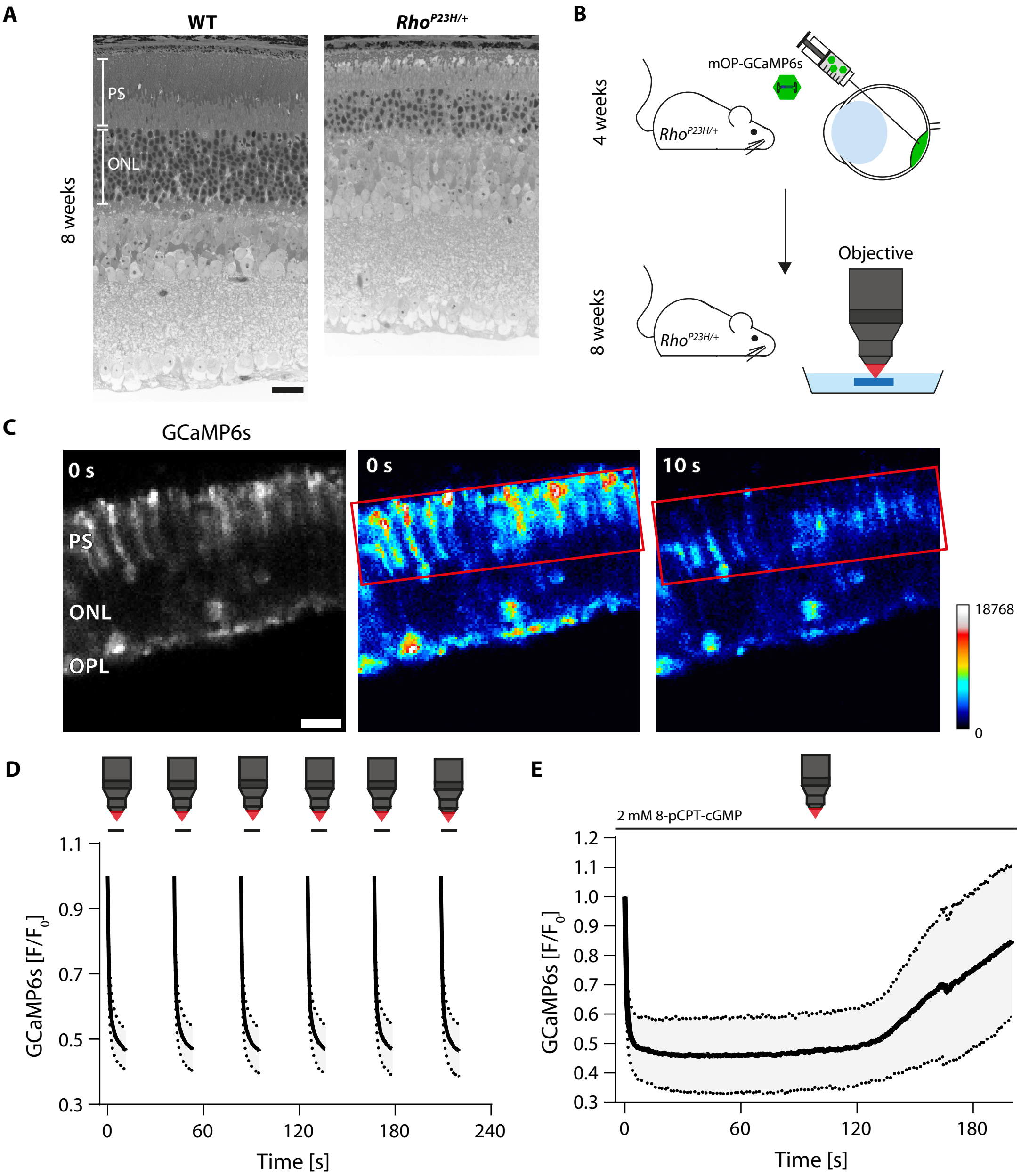

Fig. S8: Light induced response of rods in *Rho$^{P23H/+}$* mice during 2P. A, Representative images of the retinal morphology of wildtype (WT, C57B6/J) and *Rho$^{P23H/+}$* mice at 8 weeks of age. **B,** Schematic representation of the experimental timeline. Mice received a subretinal injection at 4 weeks of age. 2P of acute eye sections was performed at 8 weeks of age. **C,** Representative 2P images of acute eye sections expressing GCaMP6s in rods of *Rho$^{P23H/+}$* mice. Left: maximum intensity at 0 s. Middle and right: intensity-weighted images at 0 s (middle) and 10 s (right) of the imaging series. Red boxes mark the analyzed area (photoreceptor segments). **D,** GCaMP6s traces (mean ± SD) imaged in photoreceptor segments of *Rho$^{P23H/+}$* mice (N = 9, n = 4) during 10 s of 2P imaging followed by 30 s recovery. **E,** GCaMP6s trace (mean ± SD) of the photoreceptor segments of *Rho$^{P23H/+}$* mice (N = 5, n = 4) in the presence of 2 mM 8-pCPT-cGMP. Scale bars: 10 µm. ONL: outer nuclear layer. OPL: outer plexiform layer. PS: photoreceptor segments.

Table S1: Drugs used for 2P or 2P-FLIM experiments

Drug	Stock conc.	Solvent	Final conc.	Source, cat. no.
Sodium azide (NaN_3)	5 M	H_2O	5 mM	Sigma-Aldrich, S2002
8-pCPT-cGMP; 8- (4-Chlorophenylthio)guanosine- 3', 5'- cyclic monophosphate	20 mg	ACSF	2 mM	Enzo BioLog (Farmingdale, NY, USA), C009-50
DAB; 1,4-Dideoxy-1,4-imino-D-arabinitol hydrochloride	100 mM	H_2O	300 µM	Santa Cruz (Dallas, TX, USA), sc-220553A
AR-C155858	10 mM	DMSO	5 µM	BioTechne Torcis (Bristol, UK), 4960/1
Cytochalasin B (cytoB)	10 mM	DMSO	20 µM	BioTechne Torcis, 5474/10
Syrosingopine	10 mM	DMSO	4 µM	Merck Millipore Chemicals, SML1908
